# Isolation, Structural Elucidation, and Biological Evaluation of Pyrrole-Based Alkaloids from Sea Anemone-Associated *Streptomyces* sp. S1502

**DOI:** 10.3390/md24010051

**Published:** 2026-01-21

**Authors:** Xin Zhang, Qihong Yang, Le Zhou, Yingying Chen, Jianhua Ju, Junying Ma

**Affiliations:** 1 State Key Laboratory of Tropical Oceanography, Guangdong Key Laboratory of Marine Materia Medica, South China Sea Institute of Oceanology, Chinese Academy of Sciences, Guangzhou 510301, China; zhangxin19@mails.ucas.ac.cn (X.Z.); yychen@scsio.ac.cn (Y.C.); 2 College of Oceanology, University of Chinese Academy of Sciences, Qingdao 266400, China; 3 People’s Hospital of Longhua, Shenzhen 518109, China; mailyqh@163.com; 4 College of Pharmacy, Shenzhen Technology University, Shenzhen 518000, China; zhoule@sztu.edu.cn; 5 State Key Laboratory of Discovery and Utilization of Functional Components in Traditional Chinese Medicine, Key Laboratory of Chemical Biology (Ministry of Education), Shandong Basic Science Research Center (Pharmacy), School of Pharmaceutical Sciences, Cheeloo College of Medicine, Shandong University, Jinan 250012, China

**Keywords:** OSMAC, *Streptomyces* sp. S1502, streptopyrroles, cytotoxic activity

## Abstract

Three new pyrrole alkaloids, streptopyrroles D–F (**1**–**3**), along with four known analogs (**4**–**7**) were isolated from Sea Anemone-Associated *Streptomyces* sp. S1502 via an OSMAC (One Strain Many Compounds)-based strategy. Their structures were elucidated through comprehensive spectroscopic analyses, including HRESIMS and 1D/2D NMR experiments (COSY, HSQC, and HMBC), and further confirmed by X-ray crystallography. Biological evaluation identified streptopyrrole (**4**) as an anti-MRSA (methicillin-resistant *Staphylococcus aureus*) agent, while **4** and **6** displayed broad-spectrum cytotoxicity and good selectivity against a panel of human cancer cell lines. Notably, **4** and **6** showed particularly potent activity against the lung cancer cell lines H1299, SW1573, and A549, with IC_50_ values ranging from 5.43 to 16.24 μM. Further mechanistic investigation revealed that both compounds suppress the proliferation of lung cancer cells by inducing cell cycle arrest at the G_0_/G_1_ phase and impair metastatic potential by inhibiting migration and invasion. These findings not only expand the structural diversity of marine-derived pyrrole alkaloids but also reveal the anticancer mechanisms of **4** and **6**, highlighting their promise as active candidates for further antitumor drug development, particularly in lung cancer.

## 1. Introduction

Marine microorganisms remain an invaluable source of natural products with novel chemical structures and diverse biological activities from marine environments [1,2]. Among them, the genus *Streptomyces* represents a particularly rich resource for producing complex and bioactive secondary metabolites [3,4]. Genomic analyses indicate that each *Streptomyces* genome harbors 30–40 biosynthetic gene clusters, yet most are cryptic under standard laboratory conditions [5,6]. Genome mining has been a powerful strategy for uncovering silent biosynthetic clusters of marine microorganisms [7]. Among various activation approaches, the OSMAC strategy has proven to be highly effective in activating silent gene clusters [8], leading to the discovery of novel compounds such as mintaimycins [9], chlorobactine A [10], and scopularides [11].

Natural products discovered from *Streptomyces* are biosynthesized via major pathways such as polyketide synthase (PKS), nonribosomal peptide synthetase (NRPS), terpene synthase, and their hybrids [12,13]. Notably, PKS-NRPS hybrids yield structurally novel scaffolds with potent biological activities, such as enediynes [14], ansamycins [15], and salinosporamides [16]. Pyrrole-containing natural products, which often originate from hybrid NRPS-PKS systems, have attracted significant attention due to their broad biomedical potential. Halogenation is a critical modification that enhances their biological properties [17], as exemplified by marinopyrroles [18], armeniaspirols [19] and streptopyrroles [20]. Among these structurally diverse pyrrole derivatives from *Streptomyces*, the streptopyrrole family represents a notable class characterized by a halogenated pyrrole core. Streptopyrrole, first reported in 1998 from *Streptomyces rimosus* [20], showed antimicrobial activities against Gram-positive bacteria and histidine kinase inhibitory towards *Escherichia coli* [21]. More recently, streptopyrroles B and C, isolated from *Streptomyces zhaozhouensis* in 2023, exhibited significant antimicrobial activity against Gram-positive bacteria and cytotoxicity against several human tumor cell lines [22]. These findings not only confirm the potential of the streptopyrrole family in anti-infective and anticancer therapy but also suggest rich chemical diversity and structure-activity relationships yet to be fully explored. However, the currently known members of this family remain limited, and their complete chemical diversity, associated biological profiles, and the specific molecular mechanisms underlying their anticancer effects are far from being fully elucidated.

Therefore, the exploration of this compound class to discover novel structural analogues is of great importance. In this study, we employed an OSMAC-based strategy, guided by antimicrobial activity screening, to activate and chemically mine the sea anemone-derived strain *Streptomyces* sp. S1502. This strain was selected based on the anti-MRSA (Methicillin-resistant *Staphylococcus aureus*) activity of its extract. This effort leads to the isolation of seven streptopyrrole derivatives, including three new compounds, streptopyrroles D–F (**1**–**3**), and four known analogues (**4**–**7**). Subsequent antibacterial profiling of the purified metabolites identified streptopyrrole (**4**) as the primary contributor to the observed anti-MRSA activity of the crude extract. Furthermore, we evaluated the antitumor potential of the isolated compounds against a distinct panel of human cancer cell lines and conducted more in-depth mechanistic studies than previously reported, including investigations into cell cycle arrest, migration, and invasion. This work provides new chemical entities and a deeper mechanistic understanding to support the future development of streptopyrrole-based therapeutics. Here, we report the isolation, structural elucidation, and evaluation of the anti-MRSA and cytotoxic activities (with a preliminary mechanistic investigation) of the isolated streptopyrroles D–F (**1**–**3**) and known analogues (**4**–**7**).

## 2. Results

### 2.1. OSMAC-Guided Fermentation and Isolation of Streptopyrroles

To access the chemical diversity of *Streptomyces* sp. S1502 which was isolated from a sea anemone collected in Shenzhen Daya Bay in May 2021 [23], an OSMAC (One Strain Many Compounds) approach was carried out by cultivating the strain in eight different media. (N4, P2, TSB, Am2ab, HMT, SCAS, Am6-1, and RA) (Table S1). Comparative HPLC-UV analysis revealed a characteristic set of UV-absorbing peaks exclusively in the extract from RA medium. Subsequent antimicrobial testing confirmed that only the RA medium crude extract exhibited activity against MRSA. Therefore, scaled-up fermentation was subsequently performed in RA medium (56 L total volume) with 3% (*w*/*v*) XAD-16 resin, which provided optimal yields of the target metabolites for isolation and structural elucidation. After 7 days of cultivation at 28 °C with shaking, the resin was collected by filtration and extracted with ethanol. The combined ethanolic extracts were concentrated under reduced pressure to afford a crude residue (15.8 g).

**Figure 1:**
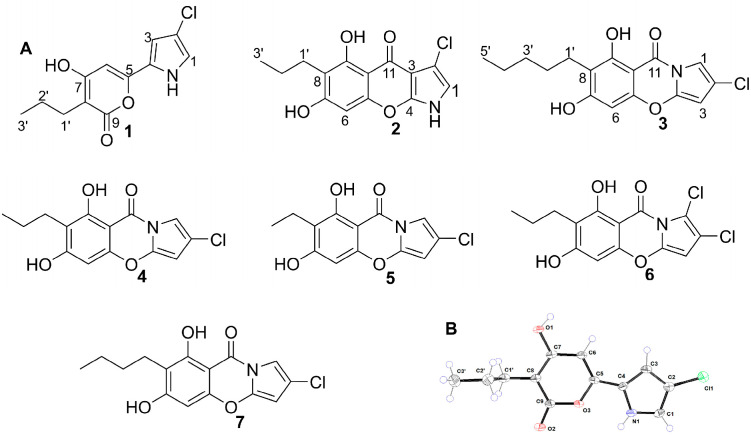
(**A**) Chemical structures of compounds **1**–**7**: streptopyrroles D–F (**1**–**3**), streptopyrrole (**4**), and known analogues (**5**–**7**). (**B**) The single-crystal structure of streptopyrroles D (**1**).

The crude extract was initially fractionated by normal-phase silica gel column chromatography using a chloroform-methanol gradient, yielding eleven primary fractions (A1–A11). Further purification of selected fractions was achieved through a combination of Sephadex LH-20 chromatography, reversed-phase ODS column chromatography, and semi-preparative HPLC. This process led to the isolation of three new metabolites **1** (7.3 mg), **2** (3.2 mg), and **3** (0.8 mg), along with four known structure derivatives: **4** (800 mg), **5** (1.8 mg), **6** (4.5 mg), and **7** (4.5 mg) (Figure 1A).

### 2.2. Structure Elucidation

**Figure 2:**
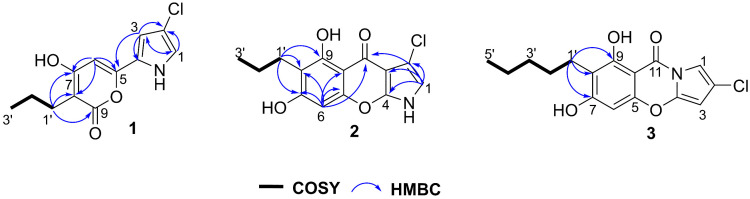
Key 2D correlations of streptopyrroles D–F (**1**–**3**).

Compound **1** was obtained as a colorless crystal. Its molecular formula was determined to be C_12_H_12_ClNO_3_ by HR-ESI-MS (*m*/*z* 254.0574 [M + H]^+^, calculated for C_12_H_13_ClNO_3_ 254.0578), corresponding to seven degrees of unsaturation. The ^1^H NMR data exhibited signals for one methyl group [*δ*_H_ 0.94 (t, *J* = 7.4 Hz, H-3′)], two methylene groups [*δ*_H_ 1.51 (m, H-2′) and *δ*_H_ 2.39 (m, H-1′)], and three olefinic methine protons [*δ*_H_ 6.61 (d, *J* = 1.6 Hz, H-1), *δ*_H_ 6.90 (d, *J* = 1.6 Hz, H-3), and *δ*_H_ 6.32 (s, H-6)]. Analysis of the ^13^C NMR and DEPT-135 spectra displayed the presence of six quaternary carbons [*δ*_C_ 125.2 (C-2), 114.9 (C-4), 153.4 (C-5), 169.1 (C-7), 103.4 (C-8), and 167.6 (C-9)], and three methine carbons [*δ*_C_ 110.1 (C-1), 120.5 (C-3), and 96.1 (C-6)] (Table 1, Figures S4–S6). The key HMBC correlations from H-1 to C-2 and C-3, along with the correlations from H-3 to C-1 and C-2, established the pyrrole ring unit (Figure 2). The downfield chemical shift in C-2 (*δ*_C_ 125.2) and the molecular formula confirmed one chlorine atom at C-2. The ^1^H-^1^H COSY correlations of H-1′/H-2′/H-3′ confirmed the presence of a propyl chain. The HMBC correlations from H-1′ to C-7, C-8, and C-9, together with those from H-6 to C-5, C-7, and C-8, defined the α-pyrone unit, which was connected to the propyl group at C-8. The key HMBC correlation from H-3 to C-5 connected the 2-chloropyrrole ring to the *α*-pyrone unit system. Furthermore, single-crystal X-ray diffraction of **1** further confirmed the connection relationship of the above fragments (Figure 1B). Thus, the structure of compound **1** was identified as displayed in Figure 1 and trivially named streptopyrrole D.

Compound **2** was obtained as a white amorphous powder and its molecular formula was established as C_14_H_12_ClNO_4_ by HR-ESI-MS *m*/*z* 292.0384 [M − H]^−^ (calcd for C_14_H_11_ClNO_4_, 292.0382), requiring nine degrees of unsaturation. The ^1^H NMR data (Table 2) revealed one methyl group [*δ*_H_ 0.95 (t, *J* = 7.4 Hz, H-3′)], two methylene groups [*δ*_H_ 1.57 (m, H-2′) and 2.61 (m, H-1′)], and two olefinic protons [*δ*_H_ 6.72 (s, H-1) and 6.38 (s, H-6)]. The ^13^C NMR and DEPT-135 spectra showed nine quaternary carbons, including one carbonyl carbon *δ*_C_ 179.6 (C-11), and two methine carbons [*δ*_C_ 113.6 (C-1) and 94.1 (C-6)] (Table 2, Figures S13–S15). The key HMBC correlations of H-1 (*δ*_H_ 6.72) to C-2 (*δ*_C_ 102.2), C-3 (*δ*_C_ 108.1), and C-4 (*δ*_C_ 150.6) and a downshifted carbon at C-2 (*δ*_C_ 102.2) identified the 2-chlorinated pyrrole ring. The ^1^H-^1^H COSY correlations of H-1′ (*δ*_H_ 2.61, m, 2H), H-2′ (*δ*_H_ 1.57, m, 2H), and H-3′ (*δ*_H_ 0.95, t, *J* = 7.4 Hz, 3H) established the presence of a propyl group in compound **2**. Furthermore, the HMBC correlations from the olefinic proton H-6 (*δ*_H_ 6.38, s) to C-5 (*δ*_C_ 155.4), C-7, C-8, and C-10 (*δ*_C_ 103.4) and the cross-peaks from H-1′ to C-7, C-8, and C-9 confirmed the presence of an aromatic ring and the connection of the propyl chain to the aromatic ring system at position C-8. The HMBC correlation from H-1 (*δ*_H_ 6.72, s) and H-6 (*δ*_H_ 6.38, s) to the carbonyl carbon C-11 (*δ*_C_ 179.6) confirmed the connection between the pyrrole ring and the aromatic ring by this group (Figure 2). Therefore, the structure of compound **2** was determined and named as streptopyrrole E.

Compound **3** was obtained as a white powder. Its molecular formula was determined as C_16_H_16_ClNO_4_ by HR-ESI-MS *m*/*z* 320.0702 [M − H]^−^ (calcd for C_16_H_15_ClNO_4_, 320.0695). The ^1^H and ^13^C NMR data of **3** were quite similar to those of the known streptopyrrole (Table 2, Figures S22–S24) [21]. The molecular formula C_16_H_16_ClNO_4_ of **3** indicated an additional C_2_H_4_ unit compared to streptopyrrole (C_14_H_12_ClNO_4_). This mass difference suggested the presence of an extended alkyl side chain in **3**. The structure of this additional moiety was confirmed by the COSY correlations of H_2_-1′/H_2_-2′/H_2_-3′/H_2_-4′/H_3_-5′ and the key HMBC correlations from H-1′ (*δ*_H_ 2.62, m) to C-7 (*δ*_C_ 165.5), C-8 (*δ*_C_ 113.7), and C-9 (*δ*_C_ 161.0). Therefore, compound **3** was characterized and assigned as streptopyrrole F.

The structures of the known compounds (**4**–**7**) were determined by comparison of their ^1^H and ^13^C NMR data with those reported in the literature. They were determined to be streptopyrrole (**4**) [21], 2-Chloro-6,8-dihydroxy-7-ethyl-9H-pyrrolo [2,1-b] [1,3] benzoxazine-9-one (**5**) [21], 1,2-Dichloro-6,8-dihydroxy-7-propyl-9H-pyrrolo [2,1-b] [1,3] benzoxazine-9-one (**6**) [21], 7-Butyl-2-chloro-6,8-hydroxy-9H-pyrrolo [2,1-b] [1,3] benzoxazine-9-one (**7**) [21]. Relevant spectroscopic data are shown in Supplementary Materials (Figures S28–S41).

### 2.3. Antibacterial Activities

The anti-MRSA activity observed in the crude RA extract of strain S1502 promoted us to identify the active constituents. The antibacterial activities of the isolated compounds (**1**–**7**) were evaluated against MRSA by the microbroth dilution method. Determination of the minimum inhibitory concentrations (MICs) revealed that streptopyrrole (**4**) was the only active metabolite, exhibiting an MIC value of 4 μg/mL. For comparison, the MIC of vancomycin (positive control) was 2 μg/mL. (Table 3). This result not only identified the active constituent responsible for the anti-MRSA effect of the crude extract but also prompted a preliminary structure-activity relationship (SAR) analysis within this compound series. Notably, the mono-chlorinated streptopyrrole (**4**) displayed stronger anti-MRSA activity than its di-chlorinated analogue (**6**). Moreover, the antibacterial potency appears to be influenced by the length of the side chain, which differs from previously reported trends for pyrrole-based antibacterial agents [22], thereby providing new insights for the design of novel antibacterial leads.

### 2.4. Cytotoxic Activities

Following the characterization of their anti-MRSA activity, the isolated streptopyrroles were subsequently evaluated for cytotoxicity against a panel of human cancer cell lines to explore their bioactive potential. The antitumor activities against a panel of human cancer cells, including hepatocellular carcinoma (HCC) cell lines (HepG2, Huh-7, BEL-7404), colorectal cancer (CRC) cell lines (HCT116, SW620, HT29), pancreatic cancer (PC) cell lines (Bxpc-3, PANC-1, MiaPaCa-2) and lung cancer (LC) cell lines (H1299, SW1573, A549), were evaluated by MTT assays, with cisplatin (DDP) was serving as a positive control. Two normal human cell lines, Beas-2B (bronchial epithelium cell) and LX2 (hepatic stellate cell) were additionally used as controls for testing compounds’ selectivity for cancer cells (Table 4). The results showed that **4** and **6** had broad-spectrum anticancer activity comparable to DDP, showing inhibitory effects across all tested cell lines, and exhibited good selectivity for killing cancer cells. Notably, **4** and **6** demonstrated remarkable activity against lung cancer cells, with IC_50_ values ranging from 10.24–15.75 μM for **4** and from 5.43 to 16.24 μM for **6**. Additionally, compound **3** showed slight cytotoxicity against colorectal cancer with IC_50_ values of 51.13–69.80 μM, while **1**, **2**, **5** and **7** exhibited negligible cytotoxicity against almost all cancer cells tested with IC_50_ values above 100 μM.

**Figure 3:**
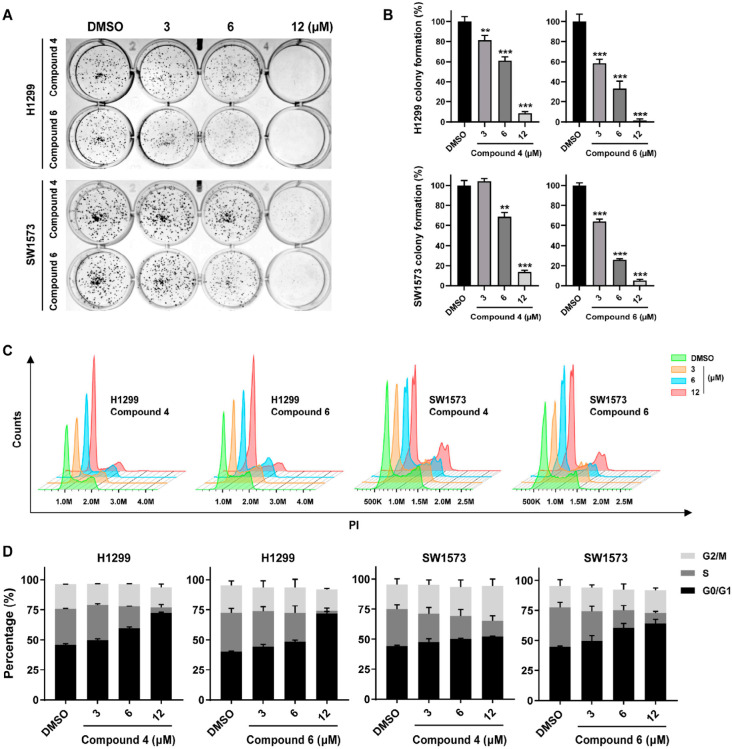
Compounds **4** and **6** markedly suppressed the proliferation of lung cancer cells by inducing the cell cycle arrest at G_0_/G_1_. (**A**) Effects of compounds **4** and **6** on the colony formation of H1299 and SW1573 cells. (**B**) Quantization for (**A**). (**C**) Effects of compounds **4** and **6** on cell cycle of H1299 and SW1573 were analyzed by flow cytometry using PI staining. (**D**) Quantization for (**C**). Data was presented as Mean ± SD, and significance was analyzed by Student’s *t*-test: **, *p* < 0.01; ***, *p* < 0.001.

**Figure 4:**
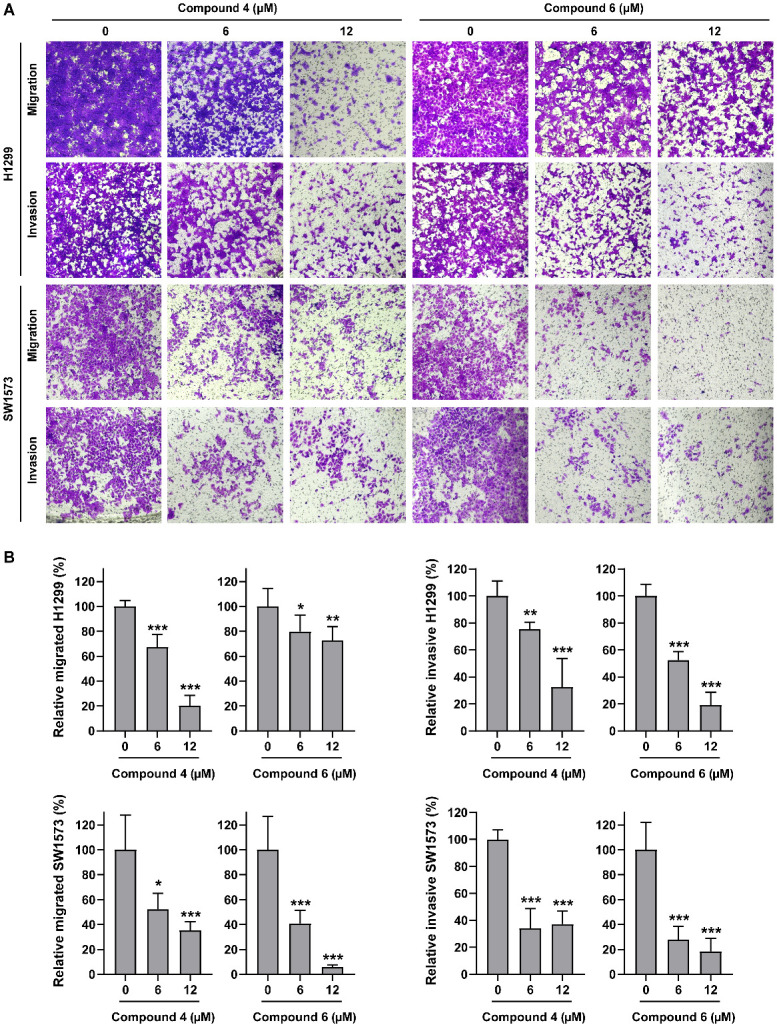
Compounds **4** and **6** significantly inhibited the migration and invasion ability in lung cancer cells. (**A**) The migration and invasion of H1299 and SW1573 influenced by compounds **4** and **6** were measured by Transwell assay. (**B**) Quantization for (**A**) by ImageJ 1.52a. Data was presented as Mean ± SD, and significance was analyzed by Student’s *t*-test: *, *p* < 0.05; **, *p* < 0.01; ***, *p* < 0.001.

Given the significant anti-lung cancer activity exhibited by compounds **4** and **6**, their effects on the proliferation and metastasis of lung cancer cell lines were further investigated in vitro. Colony formation assays demonstrated that **4** and **6** remarkably inhibited the proliferation of H1299 and SW1573 cells in a dose-dependent manner (Figure 3A,B). Cell cycle and apoptosis analysis by flow cytometry revealed that incubation with **4** or **6** efficiently and dose-dependently induced H1299 and SW1573 cells arrest at G_0_/G_1_ phase (Figure 3C,D), while exerting only slight effect on cell apoptosis (Figure S42A,B). These finding suggested that **4** and **6** suppressed the proliferation of lung cancer cells primarily by mediating cell cycle blockade rather than inducing cell apoptosis. Additionally, the result of Transwell assays showed that **4** and **6** significantly inhibited the migration and invasion ability of H1299 and SW1573 cells (Figure 4A,B). Taken together, these data indicated a promising prospect of **4** and **6** in treating lung cancer.

## 3. Material and Methods

### 3.1. General Experimental Procedures

The 1D and 2D NMR spectra were recorded on Bruker Ascend (700 MHz for ^1^H NMR). Chemical shifts are expressed in *δ* (ppm) relative to TMS as an internal standard. UV spectra were recorded on a Shimadzu UV-2600 spectrophotometer (Shimadzu, Japan). IR spectra were recorded on an IRAffinity-1 spectrometer (Shimadzu, Japan). HRESIMS data were obtained using a MaXis 4G UHR59 TOFMS spectrometer (Bruker, Germany). Analytical HPLC was performed with an Agilent 1260 HPLC system (Agilent, CA, USA), using a Phenomenex Prodigy ODS (YMC, Co., Ltd., Kyoto, Japan) column (4.6 mm × 150 mm, 5 μm). Semipreparative HPLC was performed on a Hitachi Primaide system (equipped with a 1110 isocratic pump and a 1430 DAD detector) using an C18 column (10 mm × 250 mm, 5 μm). X-ray diffraction data were collected on an XtaLAB PRO MM007HF (Rigaku, Japan) diffractometer using Cu Kα radiation.

### 3.2. Purification and Characterization of Metabolites from Streptomyces sp. S1502

For up-scaled fermentation, *Streptomyces* sp. S1502 was initially cultivated in 250 mL flasks, each containing 50 mL of RA medium, as a seed culture. After 48 h of incubation at 28 °C with shaking (200 rpm), 25 mL of this seed culture was transferred to a 1 L flask containing 200 mL of RA medium supplemented with 3% (*w*/*v*) XAD-16 resin. The production culture was then incubated under the same conditions for 7 days. Following fermentation, the XAD-16 resin was recovered from the culture broth by filtration through a mesh screen. The resin was subsequently extracted ten times with ethanol, using twice its volume for each extraction. The combined ethanolic extracts were concentrated under reduced pressure to yield a crude syrupy residue. The crude extract (15.8 g) was fractionated by normal-phase silica gel column chromatography (200–300 mesh) using a stepwise gradient of chloroform/methanol (100:0 to 0:100, *v*/*v*) to yield eleven fractions (A1–A11). Fraction A2 was further purified by Sephadex LH-20 column chromatography (methanol as eluent) to give five subfractions (B1–B5). Subtraction B1 was recrystallized to afford compound **4** (streptopyrrole, 800 mg). Subtraction B2 was separated via semi-preparative HPLC (MeCN-H_2_O, 80:20, *v*/*v*; 2.5 mL/min) to yield **5** (1.8 mg, *t*_R_ = 31 min), **6** (4.5 mg, *t*_R_ = 33 min), **7** (4.5 mg, *t*_R_ = 34 min), and **3** (0.8 mg, *t*_R_ = 37 min). Fractions A4 and A5 were combined and subjected to reversed-phase ODS column chromatography eluted with a gradient of methanol/water (10:90 to 100:0, *v*/*v*), yielding ten subfractions (C1–C10). Subtraction C4 was purified by semi-preparative HPLC (MeCN-H_2_O, 50:50, *v*/*v*; 2.5 mL/min) to give **1** (7.3 mg, *t*_R_ = 22 min). Subtraction C6 was processed under conditions (MeCN-H_2_O, 65:35, *v*/*v*; 2.5 mL/min) to afford **2** (3.2 mg, *t*_R_ = 19 min).

### 3.3. Physicochemical Properties of Isolated Compounds

Streptopyrrole D (**1**): a colorless crystal; UV (MeOH) *λ*_max_ (log ε) 203 (1.30), 247 (0.83), 346 (1.59) nm; IR *ν*_max_ 2924, 2856, 1653, 1541, 1246, 1126 cm^−1^; HRESIMS *m/z* 254.0574 [M + H]^+^ (calcd for C_12_H_13_ClNO_3_, 254.0578); ^1^H and ^13^C NMR data, see Table 1.

Streptopyrrole E (**2**): a white amorphous powder; UV (MeOH) *λ*_max_ (log ε) 239 (1.87), 293 (0.97), 334 (0.60) nm; IR *ν*_max_ 3213, 2960, 2870, 1643, 1571, 1433, 1120 cm^−1^; HRESIMS *m/z* 292.0384 [M - H]^−^ (calcd for C_14_H_11_ClNO_4_, 292.0382); ^1^H and ^13^C NMR data, see Table 2.

Streptopyrrole F (**3**): a white amorphous powder; UV (MeOH) *λ*_max_ (log ε) 204 (1.40), 215 (1.40), 295 (0.69) nm; IR *ν*_max_ 3388, 2926, 2854, 1635, 1460, 1300, 1116 cm^−1^; HRESIMS *m*/*z* 320.0702 [M - H]^−^ (calcd for C_16_H_15_ClNO_4_, 320.0695); ^1^H and ^13^C NMR data, see Table 2.

### 3.4. X-Ray Crystallography

Compound **1** was crystallized from MeOH. Suitable crystals of compound **1** was selected and recorded with an XtaLAB PRO MM007HF X-ray diffractometer equipped with an APEX II CCD using Cu Kα radiation. Crystal Data for **1**: orthorhombic, M_r_ = 253.68, space group Aea2 (no. 41), *a* = 13.0885(4) Å, *b* = 26.0043(11) Å, *c* = 6.7662(3) Å, *V* = 2302.93(16) Å^3^, *Z* = 1, *T* = 99.9(4) K, μ(Cu Kα) = 2.901 mm^−1^, *Dcalc* = 1.462 g/cm^3^, 5114 reflections measured (6.798° ≤ 2Θ ≤ 147.798°), 1756 unique (*R*_int_ = 0.0316, *R*_sigma_ = 0.0361). The final *R*_1_ was 0.0391 (I > 2σ(I)) and *wR*_2_ was 0.1206. CCDC number: 2515550.

### 3.5. Antibacterial Assays

The minimum inhibitory concentrations (MICs) against methicillin-resistant *Staphylococcus aureus* (MRSA) were determined for compounds **1**–**7** by the broth microdilution method [24]. An overnight culture of MRSA in LB broth was adjusted by visual comparison to a 0.5 McFarland standard tube, corresponding to approximately 1.5 × 10^8^ CFU/mL, and then diluted with fresh LB broth to achieve the final working concentration of nearly 5 × 10^5^ CFU/mL per well for the microbroth dilution assay. Test compounds (**1**–**7**) and vancomycin (positive control) were dissolved in DMSO and subjected to two-fold serial dilution in 96-well plates across a concentration range of 64–0.125 μg/mL. An equal volume of the bacterial inoculum was added to each well. Following incubation at 37 °C for 15 h, the MIC was defined as the lowest concentration at which no visible growth occurred. All assays were performed in three independent replicates.

### 3.6. Cell Lines and Cell Culture

All cell lines used in this study were obtained from ATCC, and cultured in DMEM or RPMI-1640 medium supplemented with 10% of FBS and 1% of penicillin-streptomycin, under the atmosphere (37 °C, 5% CO_2_). The normal immortalized human bronchial epithelium cell line Beas-2B was cultured in RPMI-1640 medium, while human hepatic stellate cell lines (LX2), hepatocellular carcinoma cell lines (HepG2, Huh-7, BEL-7404), colorectal cancer cell lines (HCT116, SW620, HT29), pancreatic cancer cell lines (Bxpc-3, PANC-1, MiaPaCa-2), and lung cancer cell lines (H1299, SW1573, A549) were cultured in DMEM medium.

### 3.7. Evaluating Cytotoxicity of Compounds Through MTT Assay

MTT assay was performed as described previously [25]. 96-well plates were seeded with cancer cells (density: 2000–3000 cells/well; volume: 200 μL), then treated the attached cells with gradient dilution of compounds for 72 h. 20 μL MTT regent (5 mg/mL) was added for additional 4 hours’ incubation at 37 °C, followed by discarding the supernatant and dissolving the blue crystal by 150 μL DMSO. Lastly, the absorbance was obtained by a microplate reader at 490 nm.

### 3.8. Colony Formation Assay

Colony formation assay was performed according to the method described previously [26]. 12-well plates were planted with 500 cells, and treated by DMSO or different doses of compounds for one week. The colony was fixed by methanol for 10–15 min when visible, then stained by the crystal violet solution for 20 min.

### 3.9. Analysis of Cell Cycle and Apoptosis

According to the relevant methods described previously [27], cancer cells (2 × 10^5^) were seeded into 6-well plates and incubated with compounds for 24 h, followed by collection and washing by cold PBS. Then, fixed cells with the pre-cold 70% ethanol overnight at −20 °C. The fixed cells were washed by cold PBS again and subjected to RNase A incubation (50 μg/mL) for 30 min in the dark at 37 °C, then stained with the propidium iodide (PI) solution (50 μg/mL) for 15 min at room temperature. For analysis of cell apoptosis, 1 × 10^5^ cells were seeded into 6-well plates and treated by compounds for 48 h. According to the protocol, cells were further collected, washed by PBS, and resuspended in the buffer (containing Annexin V-FITC and PI) for binding at room temperature (15 min, in dark). The fluorescence intensity was detected via a flow cytometer, and the data were analyzed by using FlowJo 10.

### 3.10. Transwell Assay

Transwell assays were performed in reference to the previous methods [28]. For analyzing cell invasion, the pre-thawed matrigel (356234, BD Biosciences, CA, USA) was diluted with pre-cooled FBS-free DMEM (ratio: 1:9), and added evenly into the upper chamber of the Transwell inserts (8-μm, 725301, NEST, Jiangsu, China), followed by incubation at 37 °C (0.5–1 h) for the polymerization of matrigel. Then, 4 × 10^4^ cells in FBS-free medium were seeded into the upper chambers of Transwell (note: for analyzing cell migration, the Transwell inserts do not be coated by matrigel), and the lower compartment was given 600 μL medium containing 20% FBS. After 24 h culture, the cells in the chambers were wiped off and the cells outside the chambers were fixed by methanol and stained by 0.1% crystal violet solution, followed by ImageJ analysis.

## 4. Conclusions

In this study, the OSMAC-based cultivation of *Streptomyces* sp. S1502 led to the discovery and isolation of three new pyrrole alkaloids, streptopyrroles D–F (**1**–**3**), along with four known analogs (**4**–**7**). Their structures were unequivocally determined using a combination of spectroscopic techniques and X-ray crystallography. The antibacterial assay indicated that streptopyrrole (**4**) showed activity against MRSA, with a minimum inhibitory concentration (MIC) of 4 μg/mL, compared to 2 μg/mL for vancomycin. Antitumor assessment demonstrated that **4** and **6** possess broad-spectrum cytotoxicity, particularly against lung cancer cell lines (IC_50_ = 5.43–16.24 μM). Further mechanistic investigation demonstrated that these two compounds suppress lung cancer progression through dual mechanisms: inhibiting proliferation by inducing G_0_/G_1_ phase cell cycle arrest and suppressing metastatic potential by impairing migration and invasion capacity. These findings not only enrich the structural diversity of pyrrole alkaloids from marine-derived streptomycetes but also highlight **4** and **6** as promising hit compounds for the development of novel antitumor agents, especially for non-apoptosis-dependent lung cancer treatment. However, the molecular targets underlying these effects and in vivo validation remain unknown. Future efforts will therefore prioritize target identification via computational and experimental approaches, alongside profiling key signaling proteins (e.g., cyclins, cadherins) and evaluating pharmacodynamics and pharmacokinetics in animal models.

## Figures and Tables

**Table 1:** ^13^C (175 MHz) and ^1^H (700 MHz) NMR data for compound **1** (CD_3_OD).

No.	1 (*J* in Hz)	
	*δ*_H_	*δ*_C_
1	6.61, d (1.6)	110.1, CH
2		125.2, C
3	6.90, d (1.6)	120.5, CH
4		114.9, C
5		153.4, C
6	6.32, s	96.1, CH
7		169.1, C
8		103.4, C
9		167.6, C
1′	2.39, m	26.1, CH_2_
2′	1.51, m	22.4, CH_2_
3′	0.94, t (7.4)	14.2, CH_3_

**Table 2:** ^13^C (175 MHz) and ^1^H (700 MHz) NMR data for compounds **2** and **3** (CD_3_OD).

No.	2 (*J* in Hz)		3 (*J* in Hz)	
	*δ*_H_	*δ*_C_	*δ*_H_	*δ*_C_
1	6.72, s	113.6, CH	7.21, s	105.1, CH
2		102.2, C		119.4, C
3		108.1, C	5.89, s	91.0, CH
4		150.6, C		142.6, CH
5		155.4, C		154.9, C
6	6.38, s	94.1, CH	6.31, s	94.3, C
7		162.8, C		165.5, C
8		113.4, C		113.7, C
9		161.7, C		161.0, C
10		103.4, C		93.4, C
11		179.6, C		159.8, C
1′	2.61, m	25.1, CH_2_	2.62, m	23.0, CH_2_
2′	1.57, m	23.0, CH_2_	1.53, m	29.4, CH_2_
3′	0.95, t (7.4)	14.4, CH_3_	1.35, m	23.6, CH_2_
4′			1.61, m	23.3, CH_2_
5′			0.97, m	14.4, CH_3_

**Table 3:** Minimum inhibitory concentrations (MICs, μg/mL) of compounds **1**–**7** against MRSA.

Compound	Methicillin-Resistant *Staphylococcus aureus* (MRSA)
**1**	>64
**2**	>64
**3**	>64
**4**	4
**5**	>64
**6**	>64
**7**	>64
Van	2

Van, vancomycin, as positive control.

**Table 4:** Cytotoxicity of compounds **1**–**7** in twelve cell lines of human cancers and two normal immortalized cell lines.

	Cell Lines	IC_50_ (μM)							
		DDP	1	2	3	4	5	6	7
HCC	HepG2	3.06 ± 0.77	>100	>100	79.27 ± 6.11	32.15 ± 2.03	>100	24.58 ± 2.44	>100
	Huh-7	15.49 ± 0.66	>100	>100	66.58 ± 2.60	74.9 ± 2.15	>100	35.34 ± 4.27	>100
	BEL-7404	4.39 ± 1.28	>100	>100	>100	15.88 ± 0.62	>100	22.72 ± 1.43	>100
CRC	HCT116	4.16 ± 0.43	>100	>100	55.18 ± 1.62	12.92 ± 0.74	>100	10.07 ± 0.34	>100
	SW620	9.26 ± 0.56	>100	>100	69.80 ± 0.93	20.50 ± 2.61	>100	29.06 ± 1.63	>100
	HT29	41.38 ± 3.04	>100	>100	51.13 ± 5.06	21.60 ± 0.66	>100	22.76 ± 2.42	>100
PC	Bxpc-3	3.22 ± 0.11	78.11 ± 8.18	>100	45.15 ± 1.09	3.03 ± 0.18	51.57 ± 6.80	4.27 ± 0.20	>100
	PANC-1	13.42 ± 0.87	>100	>100	>100	21.34 ± 3.09	>100	18.36 ± 2.07	>100
	MiaPaCa-2	12.30 ± 0.88	>100	>100	99.25 ± 2.80	20.33 ± 0.55	>100	28 17 ± 4.13	>100
LC	H1299	3.24 ± 0.12	>100	>100	>100	10.51 ± 0.20	>100	5.43 ± 0.40	>100
	SW1573	3.71 ± 0.79	>100	>100	>100	10.24 ± 2.60	>100	8.37 ± 1.78	>100
	A549	6.72 ± 1.41	>100	>100	65.87 ± 6.41	15.75 ± 0.77	>100	16.24 ± 0.94	>100
Normal	Beas-2B	17.77 ± 3.78	>100	>100	>100	87.06 ± 6.50	>100	96.45 ± 1.29	>100
	LX2	12.13 ± 1.02	>100	>100	>100	93.71 ± 3.02	>100	82.33 ± 2.65	>100

Cytotoxicity of compounds in human hepatocellular carcinoma (HCC), colorectal cancer (CRC), pancreatic cancer (PC), lung cancer (LC) cell lines and normal cell lines was evaluated by MTT assay. Cisplatin (DDP) was used as a positive control. Data were presented as Mean ± SD. IC_50_ values represented the concentrations of compounds caused 50% of growth inhibition.

## Data Availability

The authors declare that all relevant data supporting the findings of this study are available within the article and its Supplementary Material files or from the corresponding authors upon request. Accession Codes: Deposition Numbers 2515550 contain the supplementary crystallographic data for this paper. These data can be obtained free of charge via the joint Cambridge Crystallographic Data Centre (CCDC).

## Supplementary Materials

The following supporting information can be downloaded at https://www.mdpi.com/article/10.3390/md24010051/s1, Table S1: Compositions of culture media; Figures S1–S41: HRESIMS and NMR data of compounds **1**–**7**; Figure S42: Compounds **4** and **6** induced a slight apoptosis in lung cancer cells.
