# Supplementary material for: Isolation, Structural Elucidation, and Biological Evaluation of Pyrrole-Based Alkaloids from Sea Anemone-Associated Streptomyces sp. S1502
Source: Mar Drugs. 2026 Jan 21;24(1):51. doi: 10.3390/md24010051 (PMC12843409; doi:10.3390/md24010051)
Supplement: Supplementary file 1 [file marinedrugs-24-00051-s001.zip › marinedrugs-4065573-supplementary.pdf]

Supporting Information

Isolation, Structural Elucidation, and Biological Evaluation of Pyrrole-Based Alkaloids from Sea Anemone-Associated *Streptomyces* sp. S1502

Xin Zhang[1, 2, *], Qihong Yang[3, *], Le Zhou[4], Yingying Chen[1], Jianhua Ju[1 2 5 #] and Junying Ma[1 2 #]

Contents

Table S1. Compositions of culture media. 3
Figure S1. HRESIMS spectrum of **1**. 3
Figure S2. IR spectrum of **1**. 3
Figure S3. UV spectrum of **1**. 4
Figure S4. ^{1}H NMR (700 MHz, CD_3OD) spectrum of **1**. 4
Figure S5. ^{13}C NMR (175 MHz, CD_3OD) spectrum of **1**. 5
Figure S6. DEPT 135 (175 MHz, CD_3OD) spectrum of **1**. 6
Figure S7. HSQC spectrum of **1**. 6
Figure S8. HMBC spectrum of **1**. 6
Figure S9. ^{1}H-^{1}H COSY spectrum of **1**. 7
Figure S10. HRESIMS spectrum of **2**. 7
Figure S11. IR spectrum of **2**. 8
Figure S12. UV spectrum of **2**. 8
Figure S13. ^{1}H NMR (700 MHz, CD_3OD) spectrum of **2**. 9
Figure S14. ^{13}C NMR (175 MHz, CD_3OD) spectrum of **2**. 9
Figure S15. DEPT 135 (175 MHz, CD_3OD) spectrum of **2**. 10
Figure S16. HSQC spectrum of **2**. 10
Figure S17. HMBC spectrum of **2**. 11
Figure S18. ^{1}H-^{1}H COSY spectrum of **2**. 11
Figure S19. HRESIMS spectrum of **3**. 12
Figure S20. IR spectrum of **3**. 12
Figure S21. UV spectrum of **3**. 12
Figure S22. ^{1}H NMR (700 MHz, CD_3OD) spectrum of **3**. 13
Figure S23. ^{13}C NMR (175 MHz, CD_3OD) spectrum of **3**. 13
Figure S24. DEPT 135 (175 MHz, CD_3OD) spectrum of **3**. 14
Figure S25. HSQC spectrum of **3**. 14
Figure S26. HMBC spectrum of **3**. 15

Figure S27. ^{1}H-^{1}H COSY spectrum of **3**..15
Figure S28. ^{1}H NMR (500 MHz, CD_3OD) spectrum of **4**. 16
Figure S29. ^{13}C NMR (125 MHz, CD_3OD) spectrum of **4**. 16
Figure S30. HRESIMS spectrum of **5**...17
Figure S31. ^{1}H NMR (700 MHz, CD_3OD) spectrum of **5**. 17
Figure S32. ^{13}C NMR (175 MHz, CD_3OD) spectrum of **5**. 18
Figure S33. DEPT 135 (175 MHz, CD_3OD) spectrum of **5**.......................................18
Figure S34. HRESIMS spectrum of **6**...19
Figure S35. ^{1}H NMR (700 MHz, CD_3OD) spectrum of **6**. 19
Figure S36. ^{13}C NMR (175 MHz, CD_3OD) spectrum of **6**. 20
Figure S37. DEPT 135 (175 MHz, CD_3OD) spectrum of **6**.......................................20
Figure S38. HRESIMS spectrum of **7**...21
Figure S39. ^{1}H NMR (700 MHz, CD_3OD) spectrum of **7**. 21
Figure S40. ^{13}C NMR (175 MHz, CD_3OD) spectrum of **7**. 22
Figure S41. DEPT 135 (175 MHz, CD_3OD) spectrum of **7**.......................................22
Figure S42. Compounds **4** and **6** induced a slight apoptosis in lung cancer cells.. 23

**N4**	Soluble Starch 15 g, Fish Peptone 8 g, Bacteriological Peptone 5 g, Glycerol 8 g, KBr 0.2 g, Sea Salt 30 g, $CaCO_3$ 2 g
**P2**	Malt Extract 10 g, Glucose 4 g, Yeast Extract 5 g, Sea Salt 30 g, $CaCO_3$ 2 g
**TSB**	Tryptic Soy Broth 30 g
**AM2ab**	Soluble Starch 5 g, Glucose 20 g, Yeast Powder 2 g, Bacteriological Peptone 2 g, Soybean Meal 5 g, KH_2PO_4 0.5 g, $MgSO_4 \cdot 7H_2O$ 0.5 g, NaCl 4 g, Sea Salt 30 g, $CaCO_3$ 2 g
**HMT**	Fish Meal 10 g, Yeast Extract 5 g, Glycerol 20 g, Sea Salt 30 g, $CaCO_3$ 5 g
**SCAS**	Soluble Starch 40 g, Acid-hydrolyzed Casein 5 g, K_2HPO_4 0.5 g, $MgSO_4 \cdot 7H_2O$ 0.5 g, $FeSO_4 \cdot 7H_2O$ 0.1 g, Sea Salt 30 g, $CaCO_3$ 2 g
**AM6-1**	Starch 20 g, Glycerol 10 g, Yeast Extract 5 g, Sea Salt 30 g, $CaCO_3$ 5 g
**RA**	Soluble Starch 20 g, Corn Meal 5 g, Glucose 10 g, Malt Extract 10 g, Maltose 10 g, Trace Elements 100 µL, Sea Salt 30 g, $CaCO_3$ 2 g

Table S1. Compositions of culture media (N4, P2, TSB, AM2ab, HMT, SCAS, AM6-1, and RA). All media were prepared at a volume of 1 L and adjusted to pH 7.2–7.6.

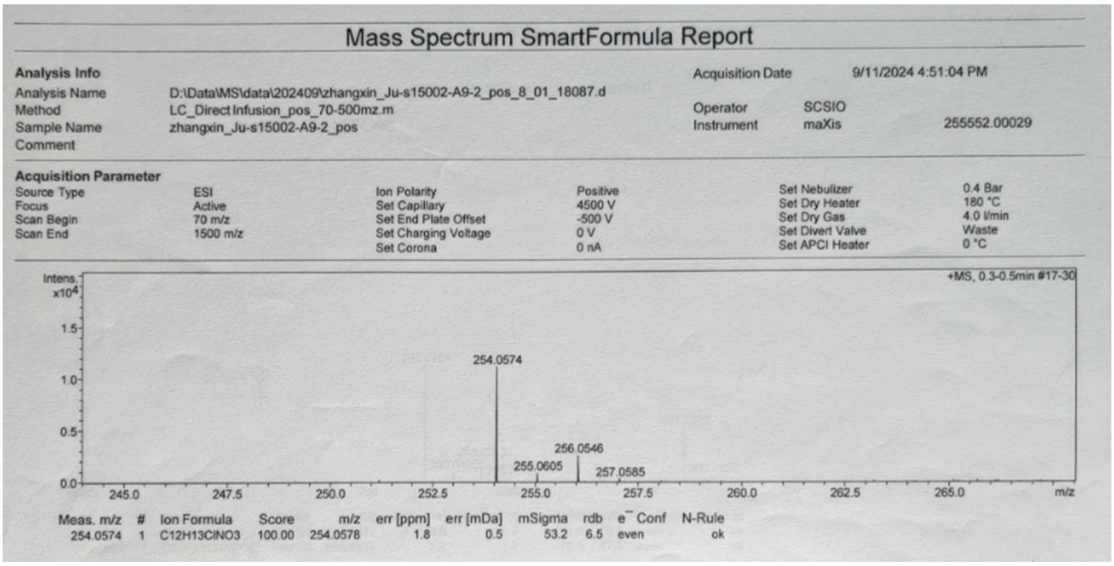

Figure S1. HRESIMS spectrum of **1**.

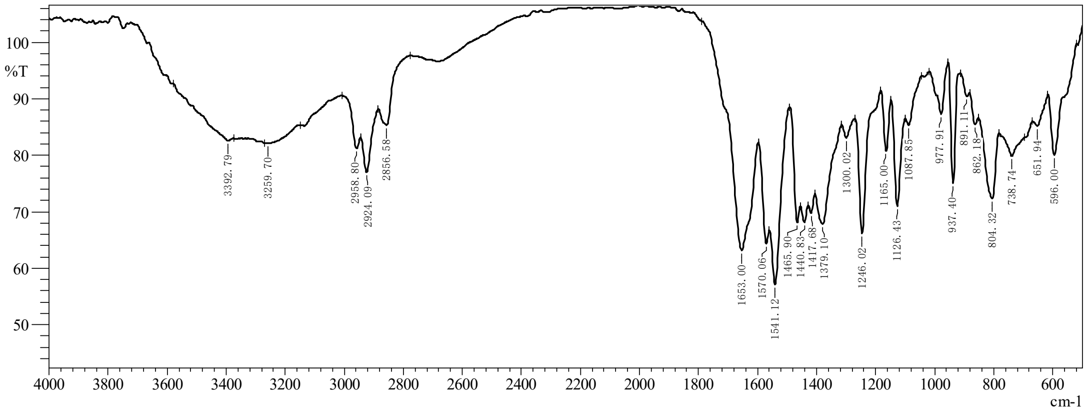

Figure S2. IR spectrum of **1**.

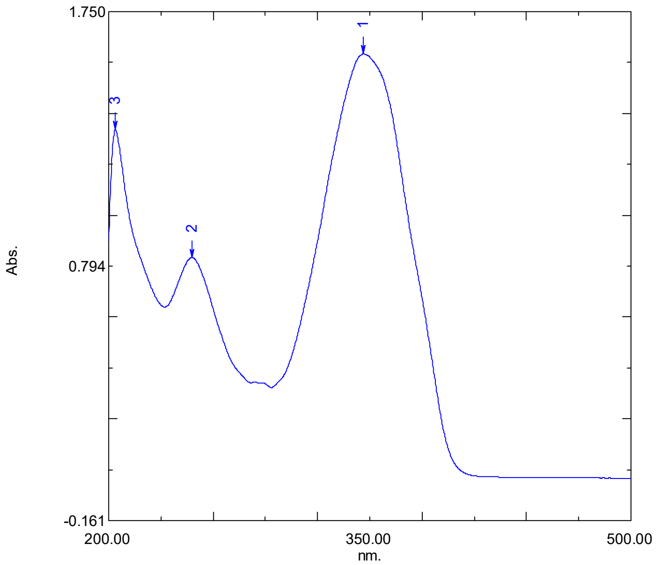

Figure S3. UV spectrum of **1**.

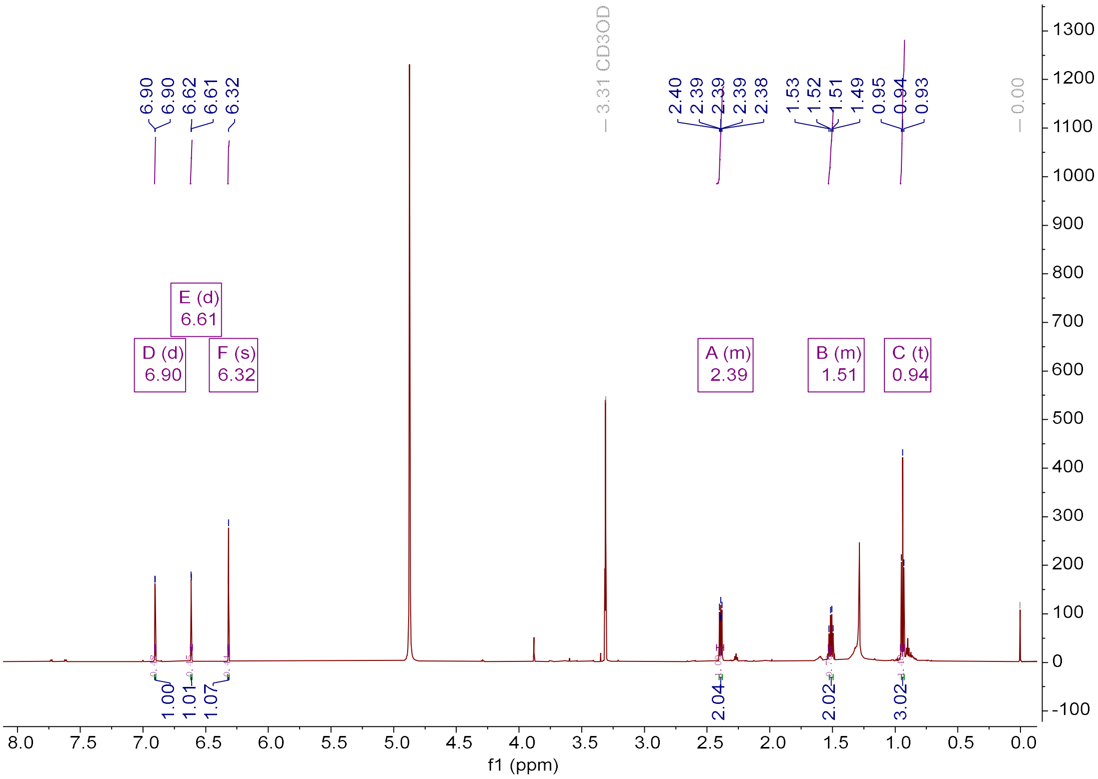

Figure S4. ^{1}H NMR (700 MHz, CD_3OD) spectrum of **1**.

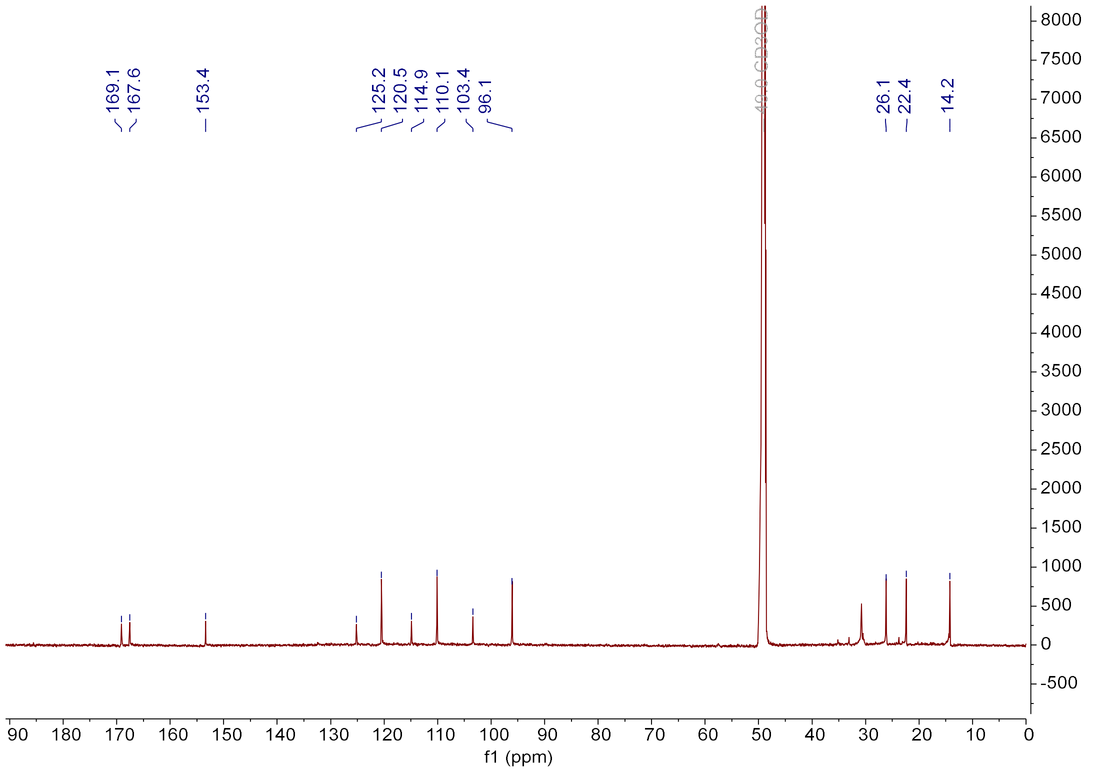

Figure S5. ^{13}C NMR (175 MHz, CD_3OD) spectrum of **1**.

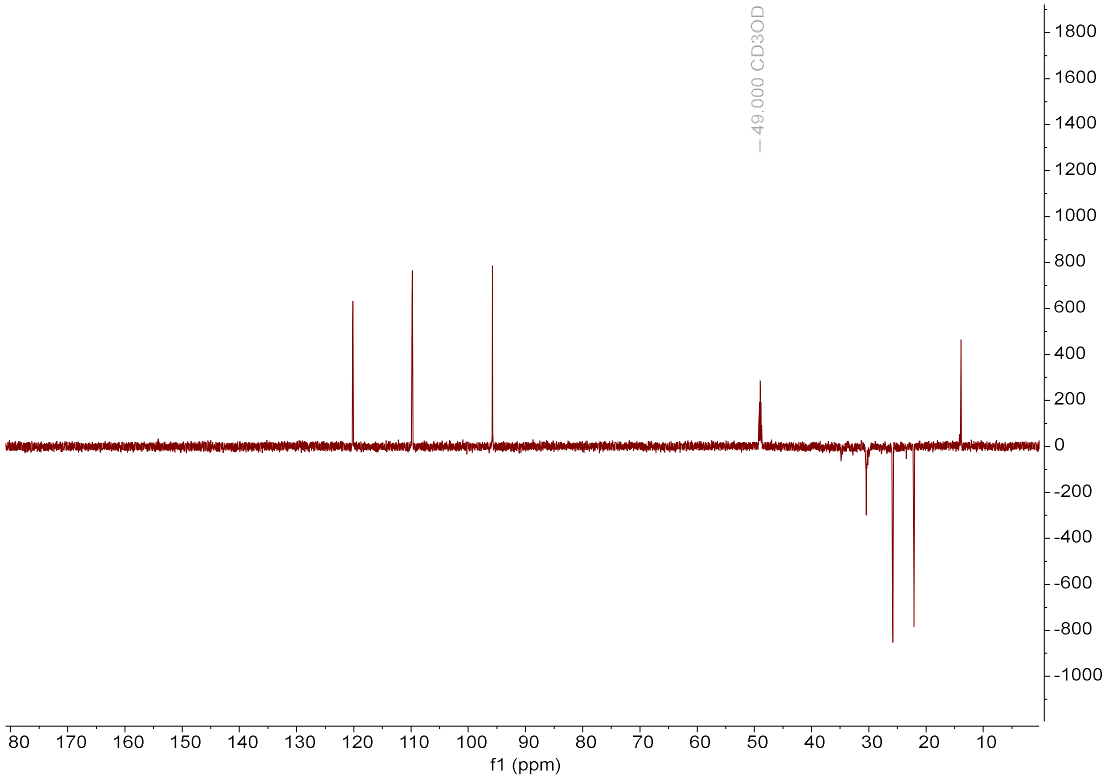

Figure S6. DEPT 135 (175 MHz, CD_3OD) spectrum of **1**.

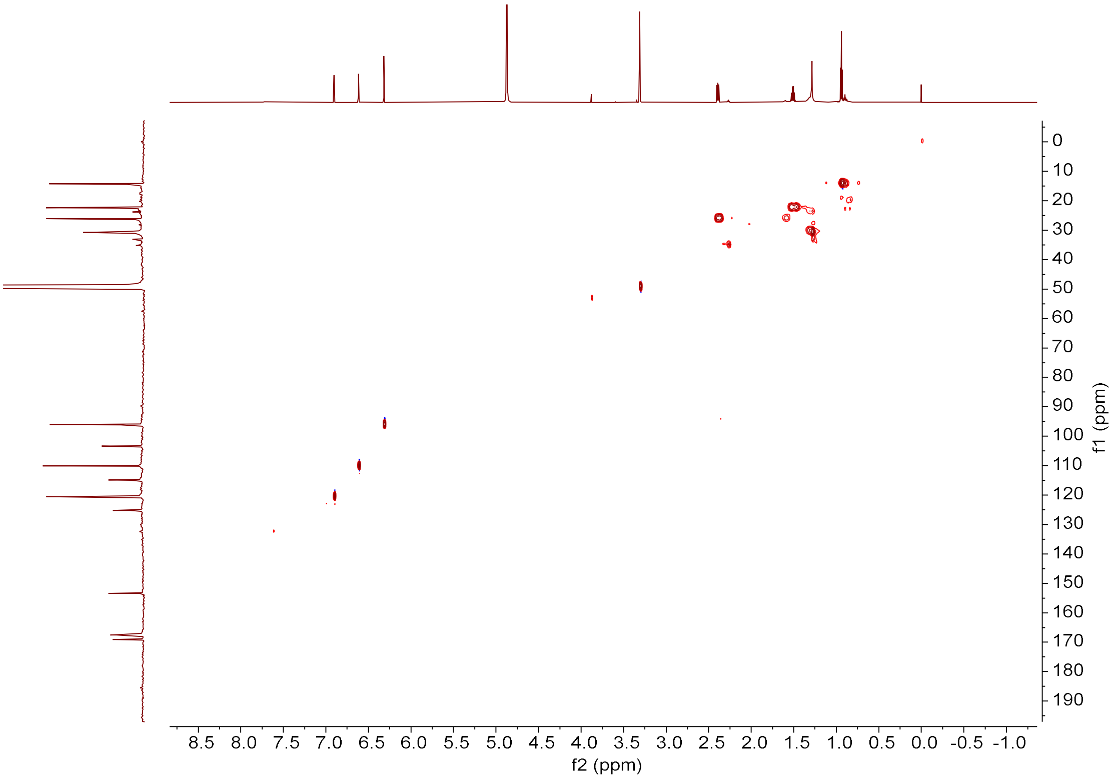

Figure S7. HSQC spectrum of **1**.

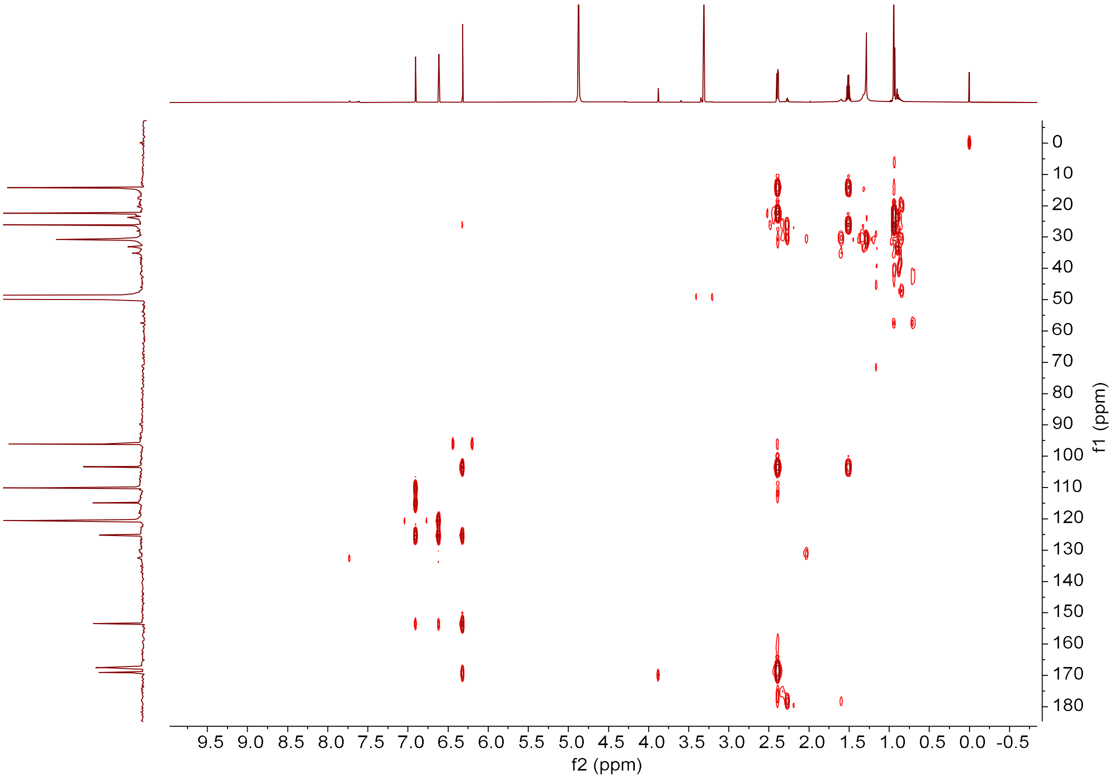

Figure S8. HMBC spectrum of **1**.

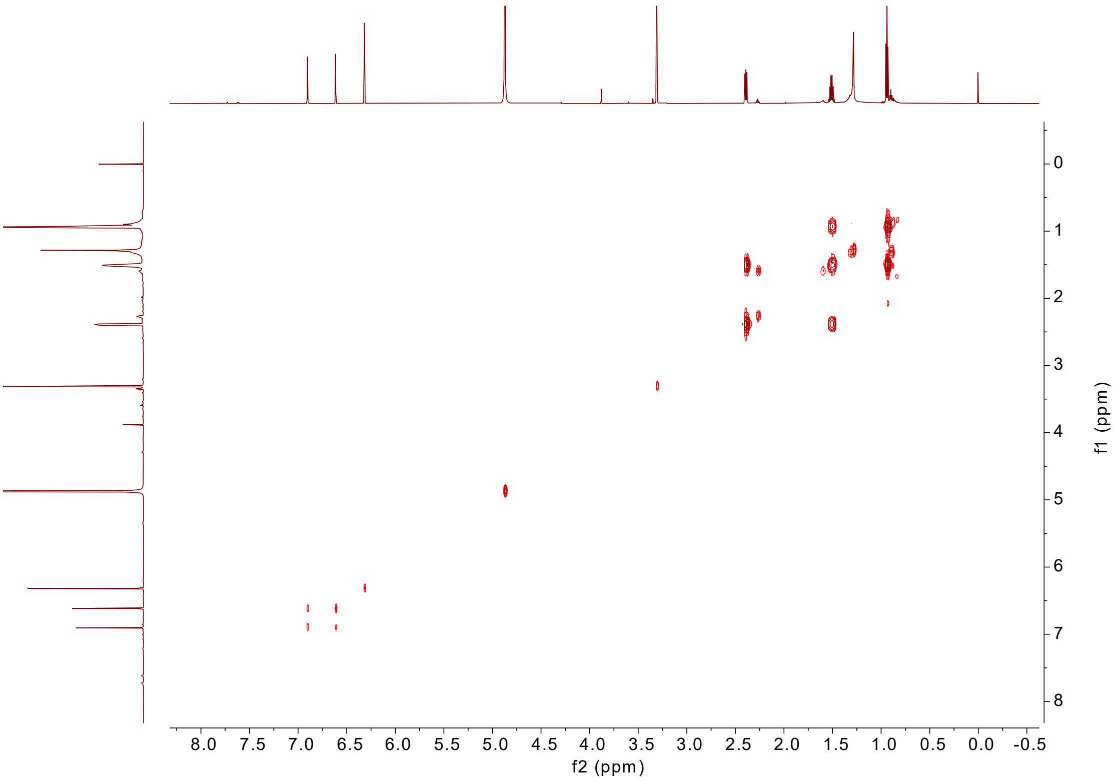

Figure S9. ^{1}H-^{1}H COSY spectrum of **1**.

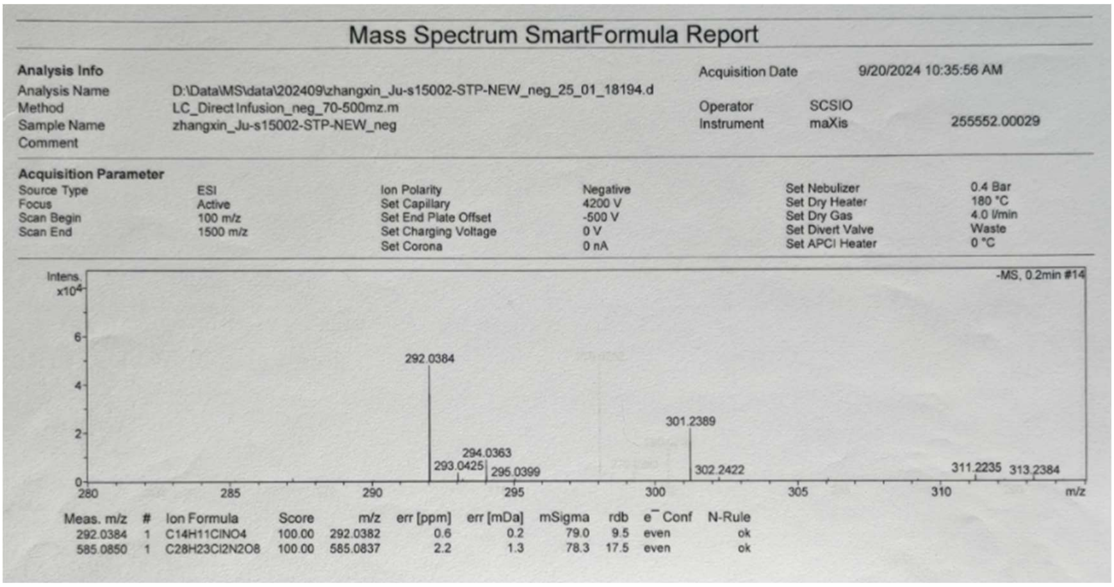

Mass Spectrum SmartFormula Report

Analysis Info

Analysis Name D:\Data\MS\data\202409\zhangxin_Ju-s15002-STP-NEW_neg_25_01_18194.d
Method LC_Direct Infusion_neg_70-500mz.m
Sample Name zhangxin_Ju-s15002-STP-NEW_neg
Comment

Acquisition Date 9/20/2024 10:35:56 AM
Operator SCSIO
Instrument maXis 255552.00029

Acquisition Parameter

Source Type	ESI	Ion Polarity	Negative	Set Nebulizer	0.4 Bar
Focus	Active	Set Capillary	4200 V	Set Dry Heater	180 °C
Scan Begin	100 m/z	Set End Plate Offset	-500 V	Set Dry Gas	4.0 l/min
Scan End	1500 m/z	Set Charging Voltage	0 V	Set Divert Valve	Waste
		Set Corona	0 nA	Set APCI Heater	0 °C

Meas. m/z	#	Ion Formula	Score	m/z	err [ppm]	err [mDa]	mSigma	rdb	e¯ Conf	N-Rule
292.0384	1	C14H11ClNO4	100.00	292.0382	0.6	0.2	79.0	9.5	even	ok
585.0850	1	C28H23Cl2N2O8	100.00	585.0837	2.2	1.3	78.3	17.5	even	ok

Figure S10. HRESIMS spectrum of **2**

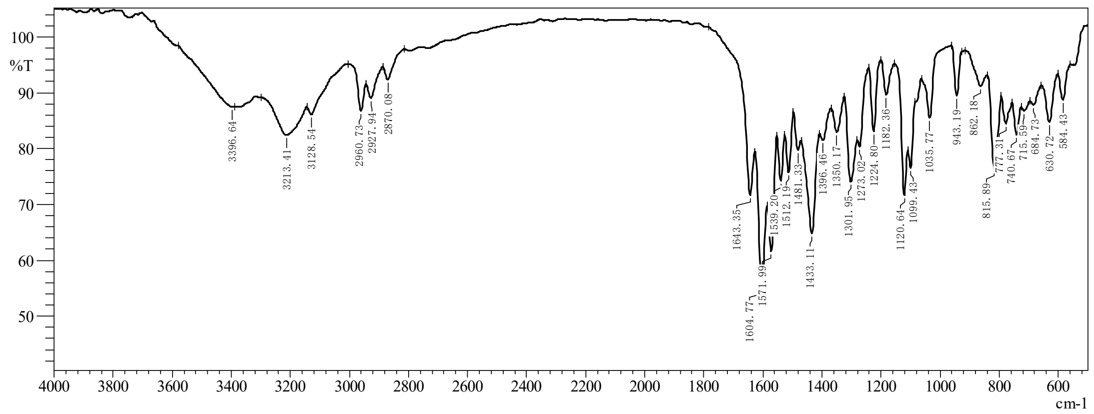

Figure S11. IR spectrum of **2**.

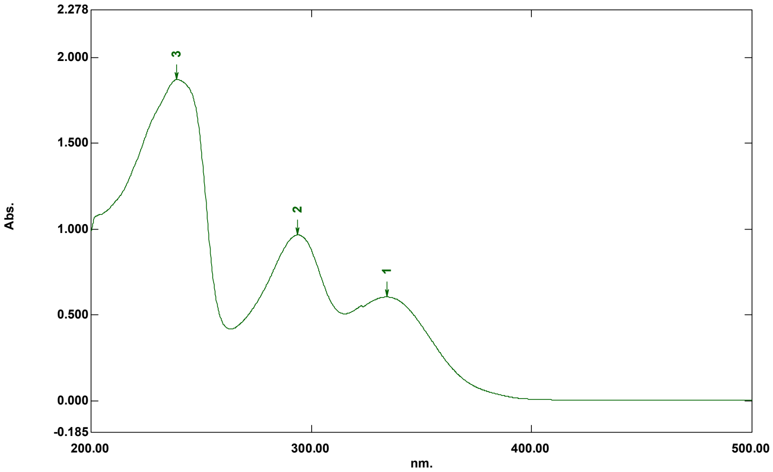

Figure S12. UV spectrum of **2**.

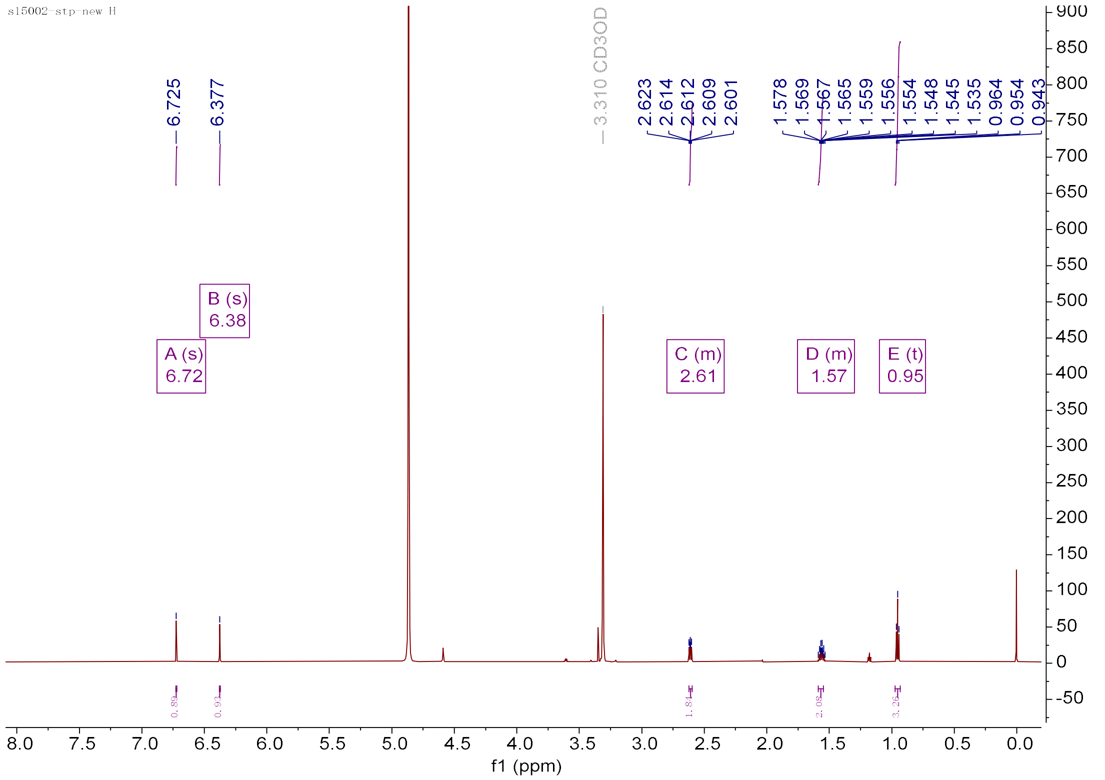

Figure S13. ^{1}H NMR (700 MHz, CD_3OD) spectrum of **2**.

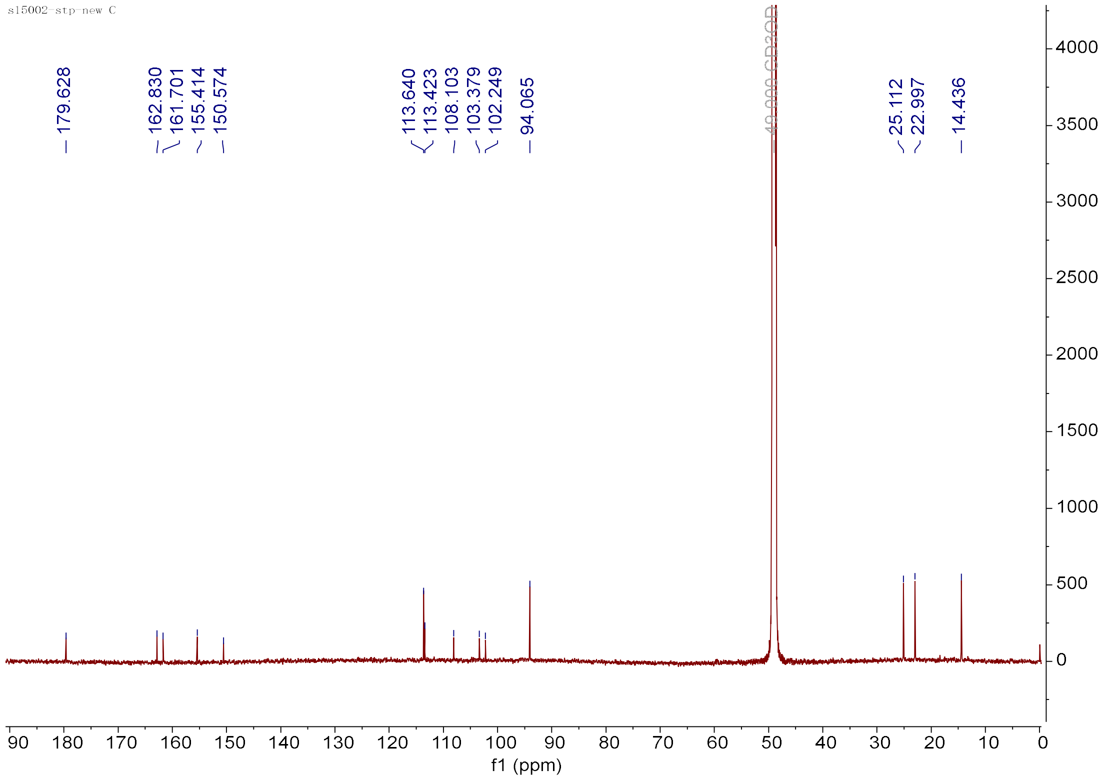

Figure S14. ^{13}C NMR (175 MHz, CD_3OD) spectrum of **2**.

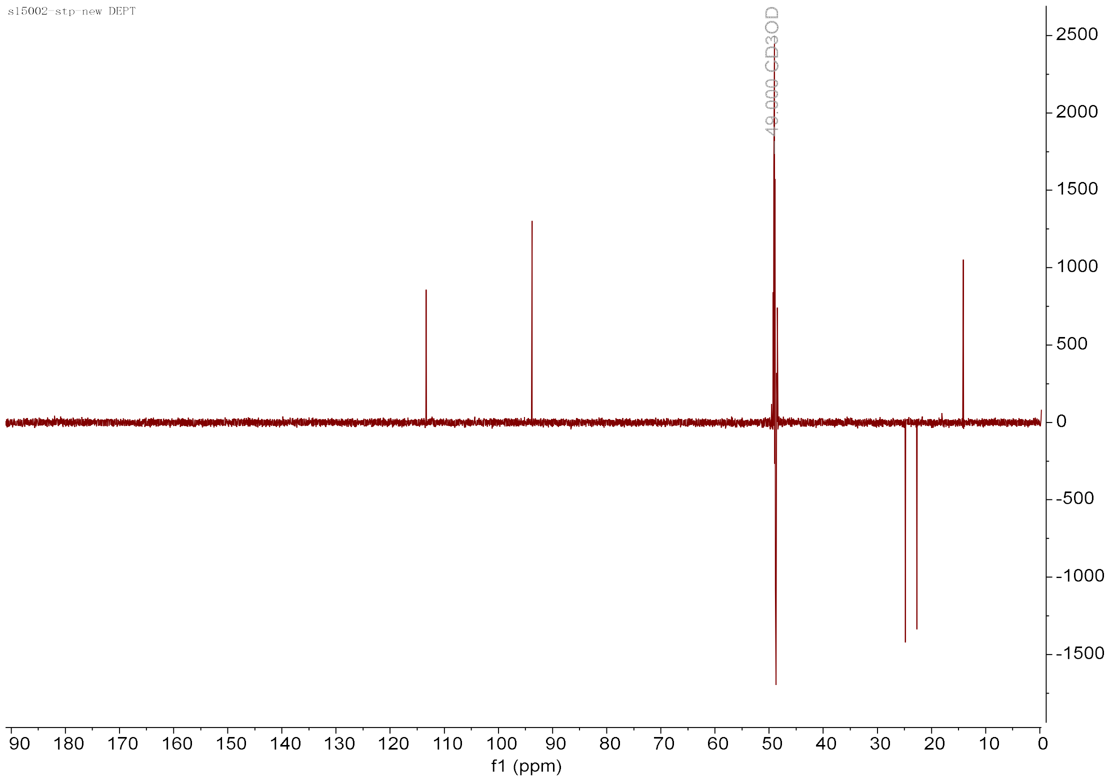

Figure S15. DEPT 135 (175 MHz, CD_3OD) spectrum of **2**.

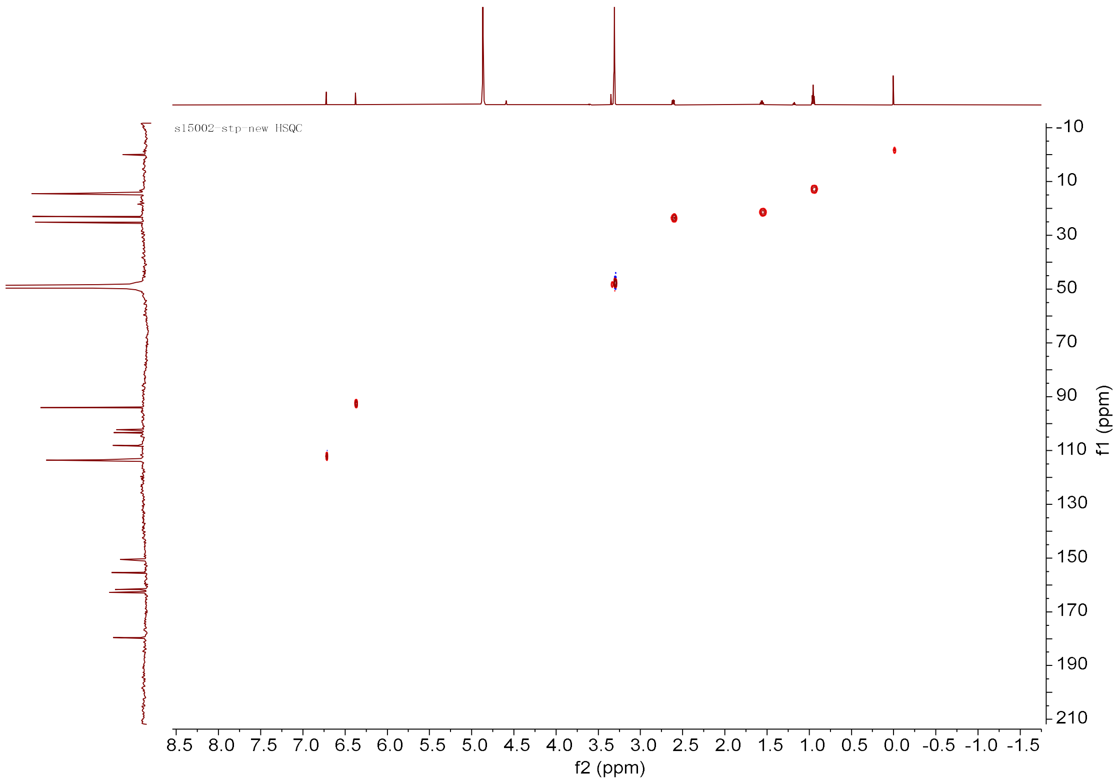

Figure S16. HSQC spectrum of **2**.

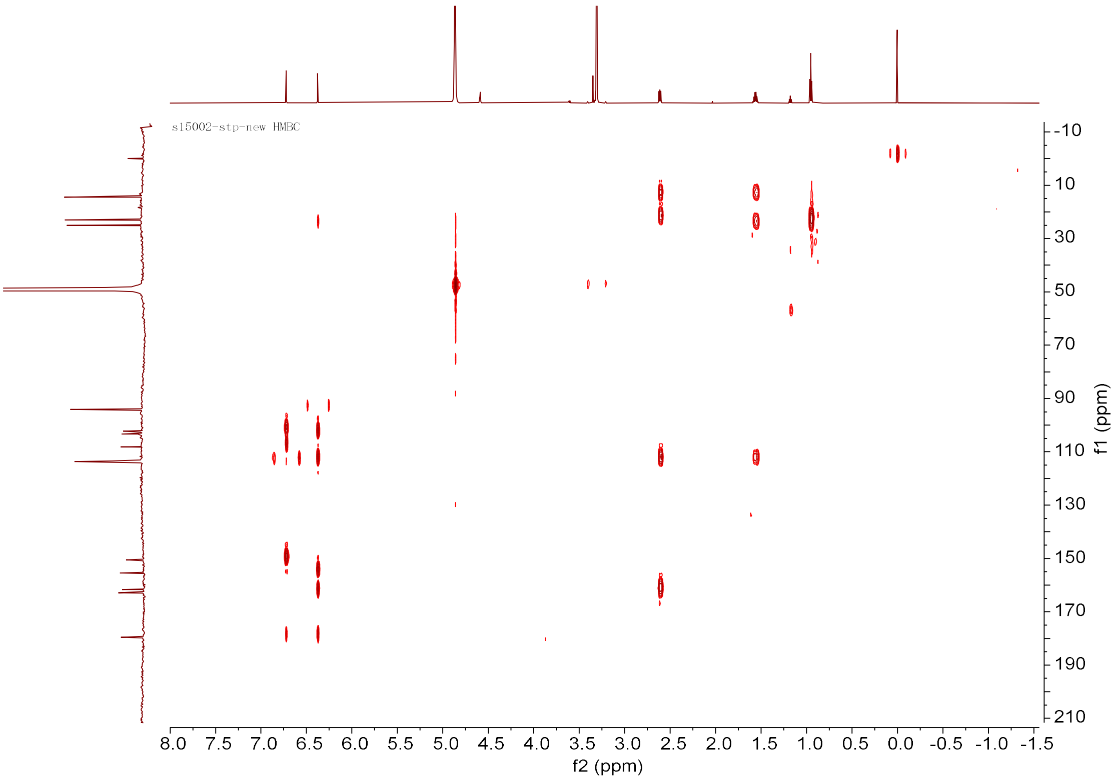

Figure S17. HMBC spectrum of **2**.

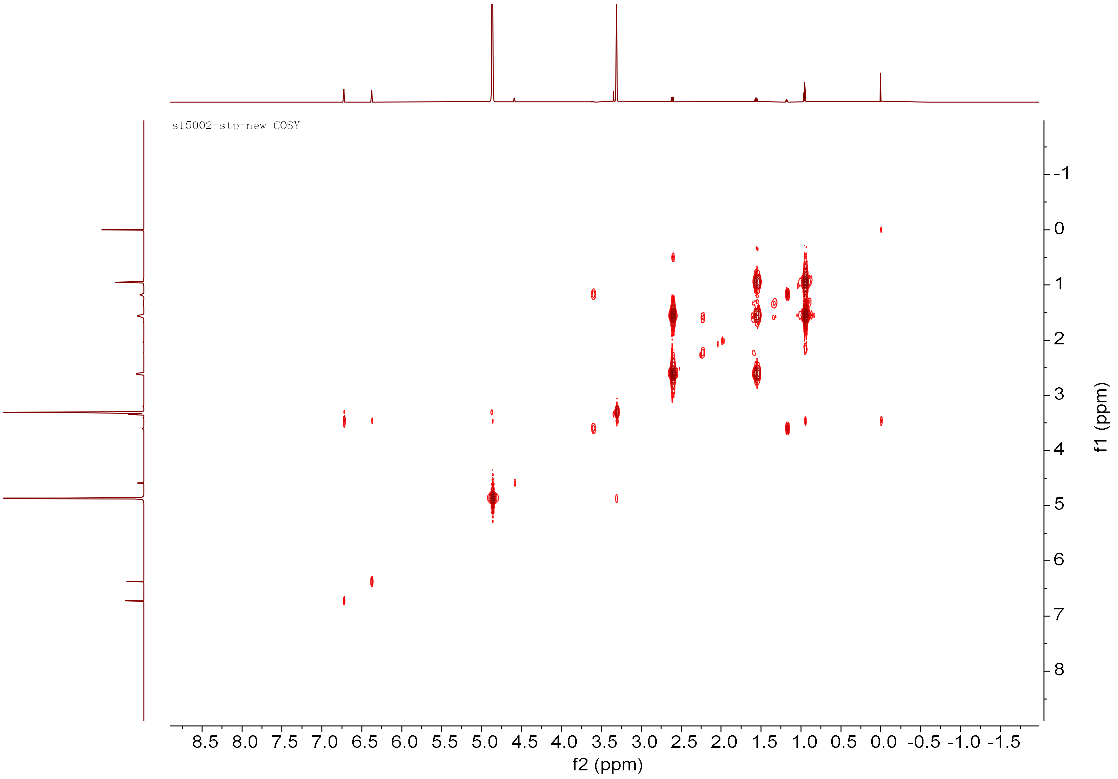

Figure S18. ^{1}H-^{1}H COSY spectrum of **2**.

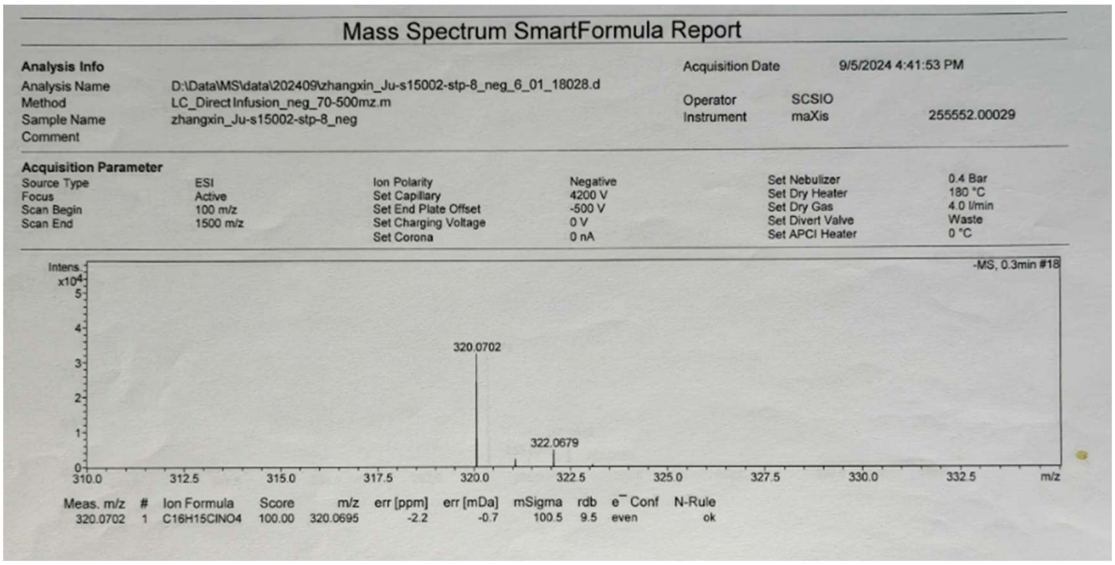

Figure S19. HRESIMS spectrum of **3**

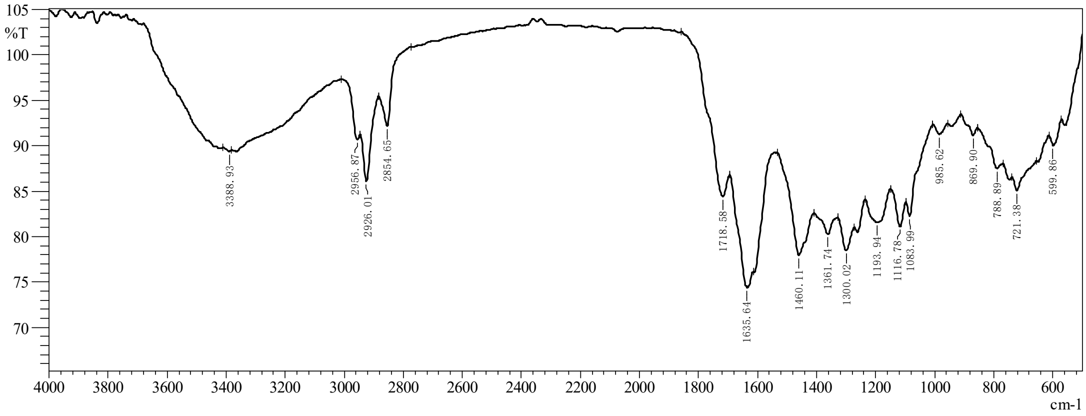

Figure S20. IR spectrum of **3**.

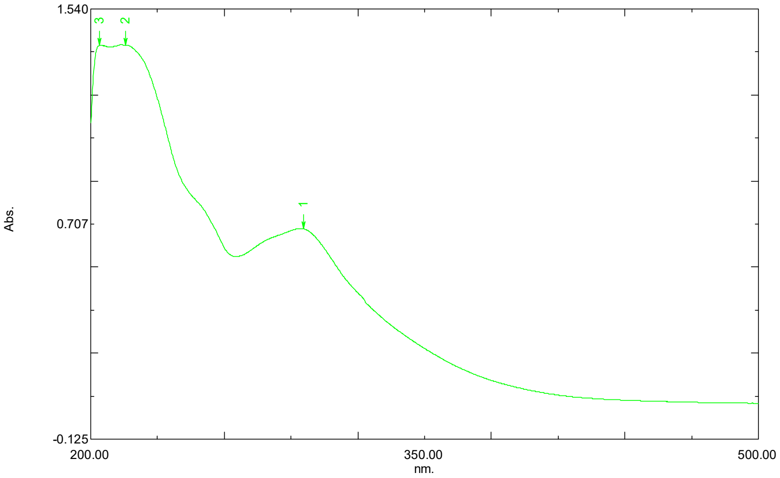

Figure S21. UV spectrum of **3**.

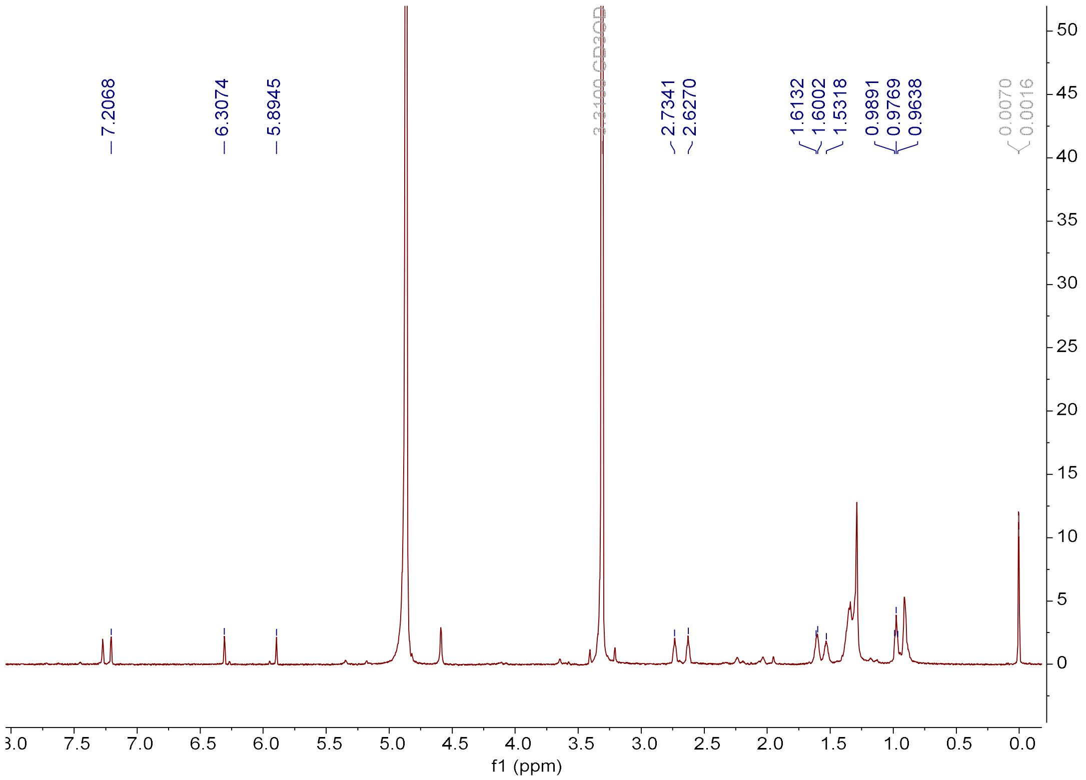

Figure S22. ^{1}H NMR (700 MHz, CD_3OD) spectrum of **3**.

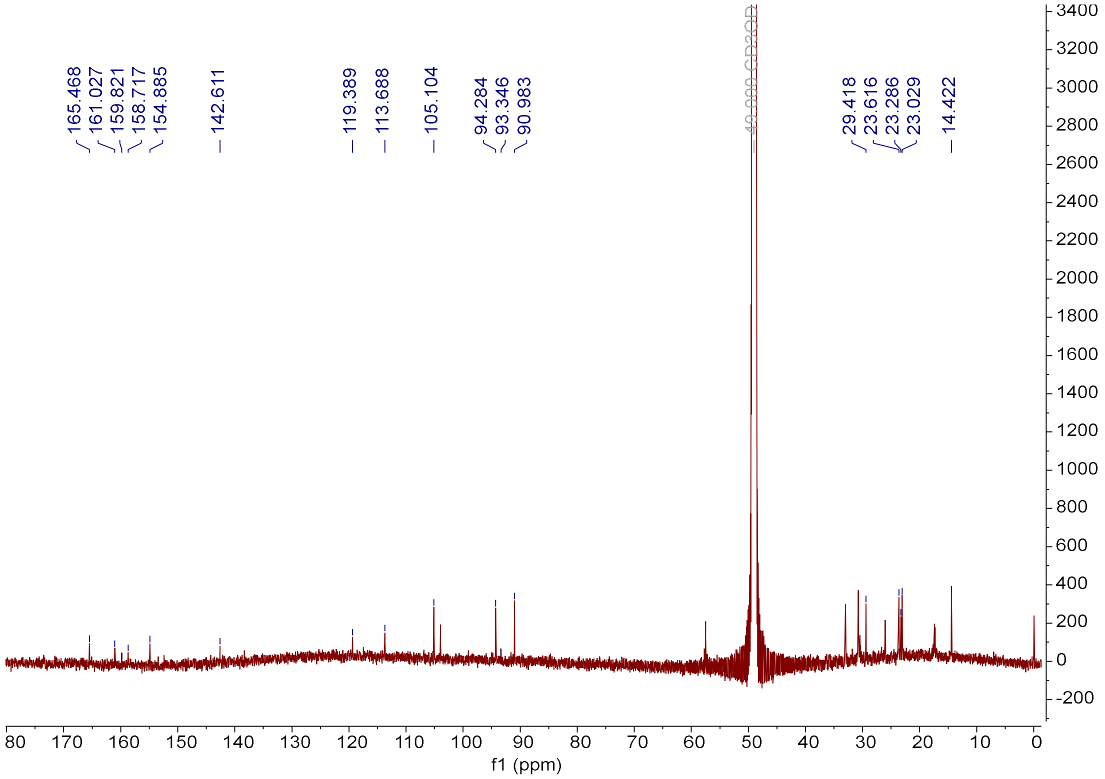

Figure S23. ^{13}C NMR (175 MHz, CD_3OD) spectrum of **3**.

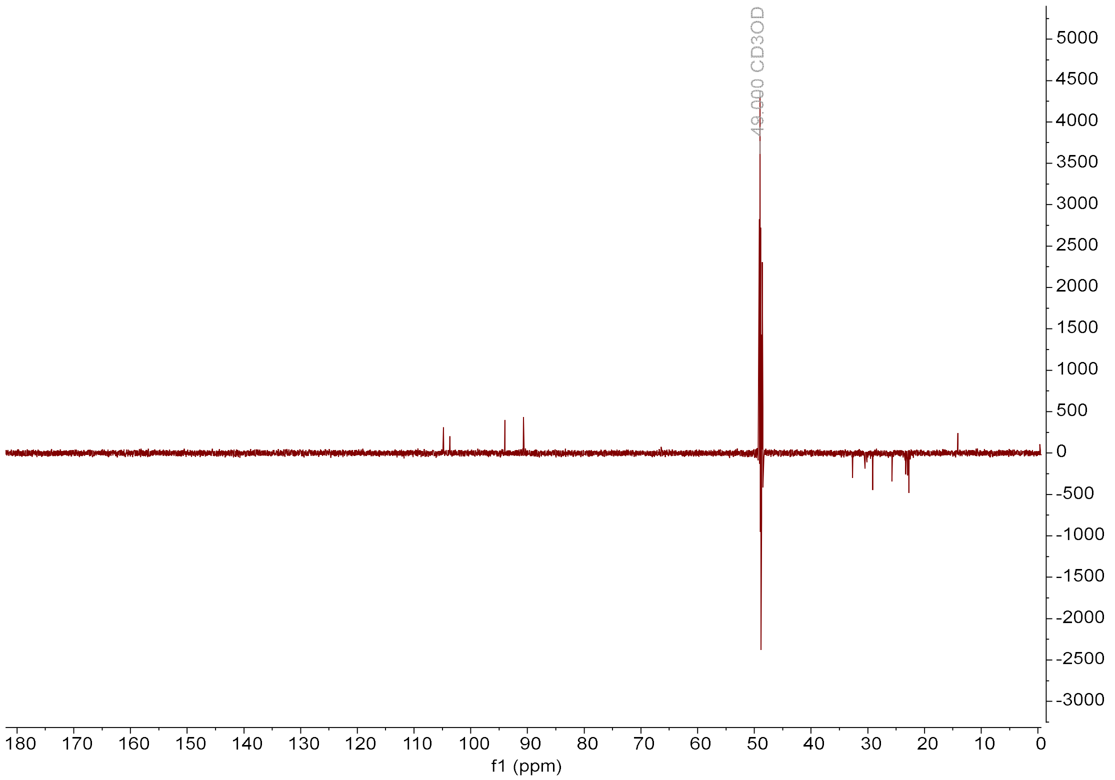

Figure S24. DEPT 135 (175 MHz, CD_3OD) spectrum of **3**.

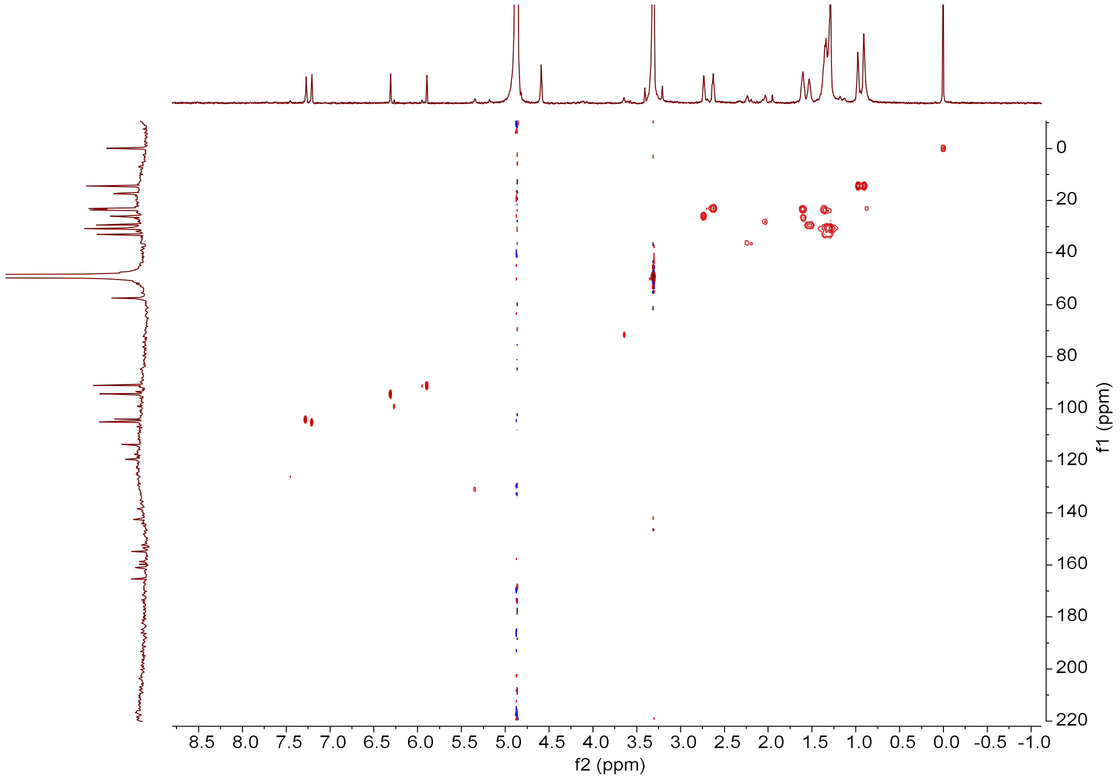

Figure S25. HSQC spectrum of **3**.

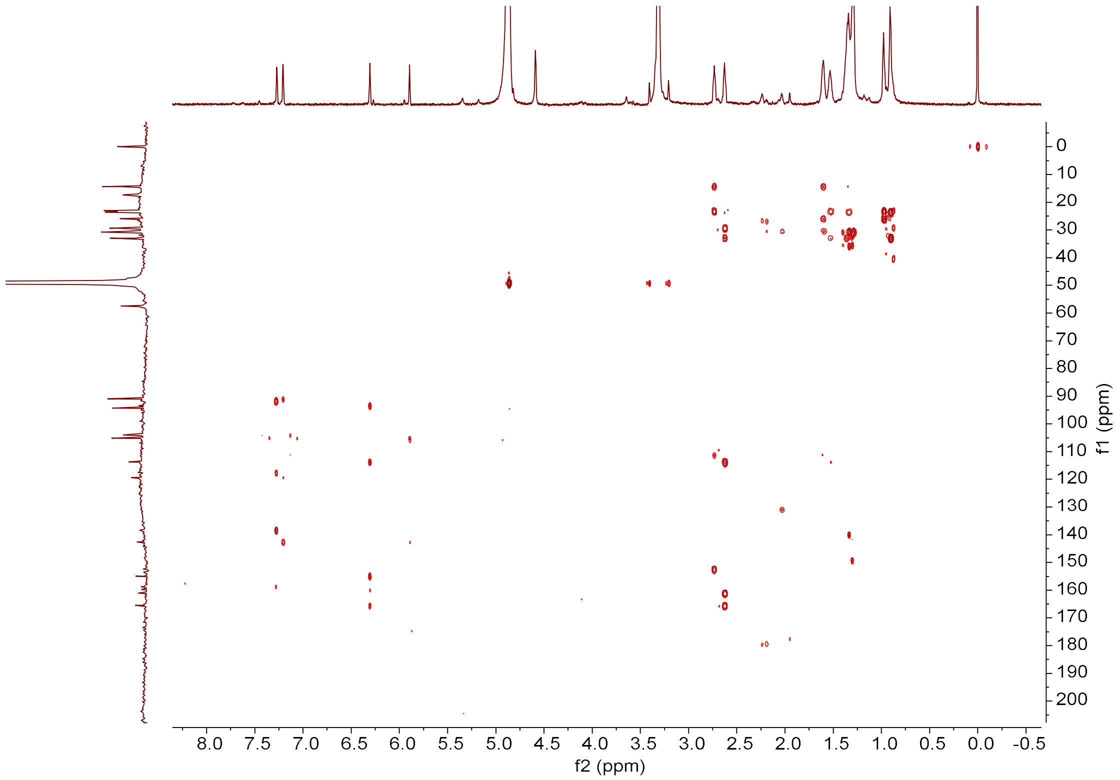

Figure S26. HMBC spectrum of **3**.

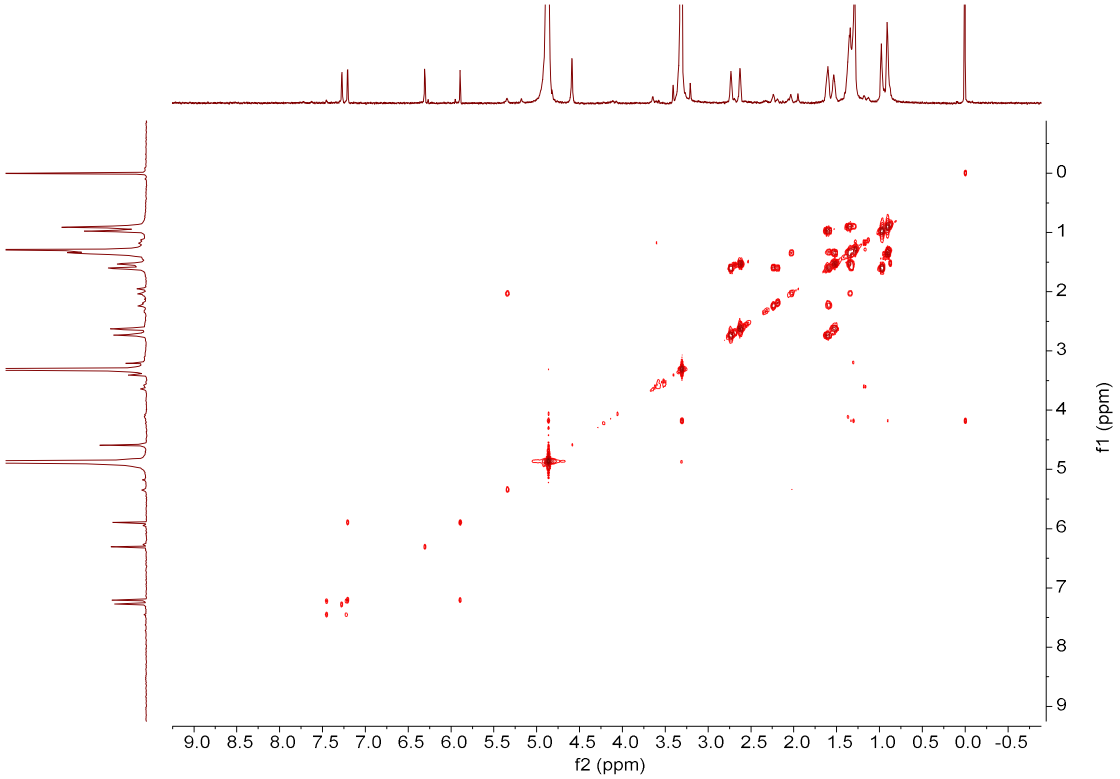

Figure S27. ¹H-¹H COSY spectrum of **3**.

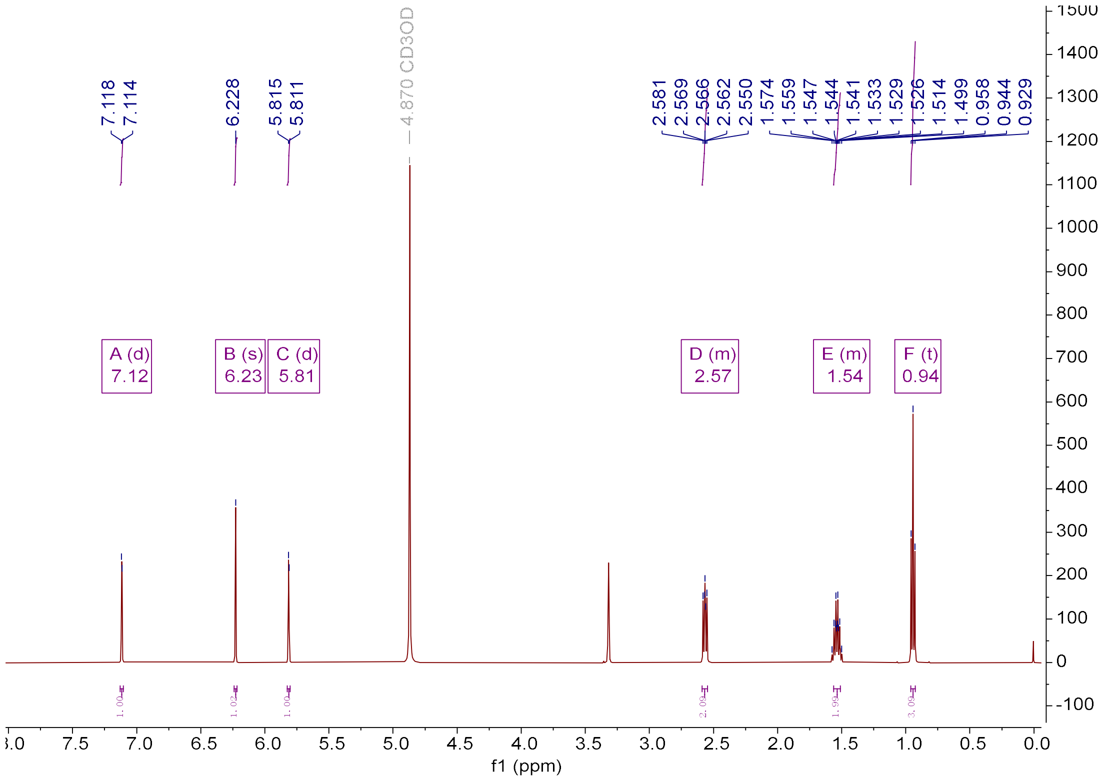

Figure S28. ^{1}H NMR (500 MHz, CD_3OD) spectrum of **4**.

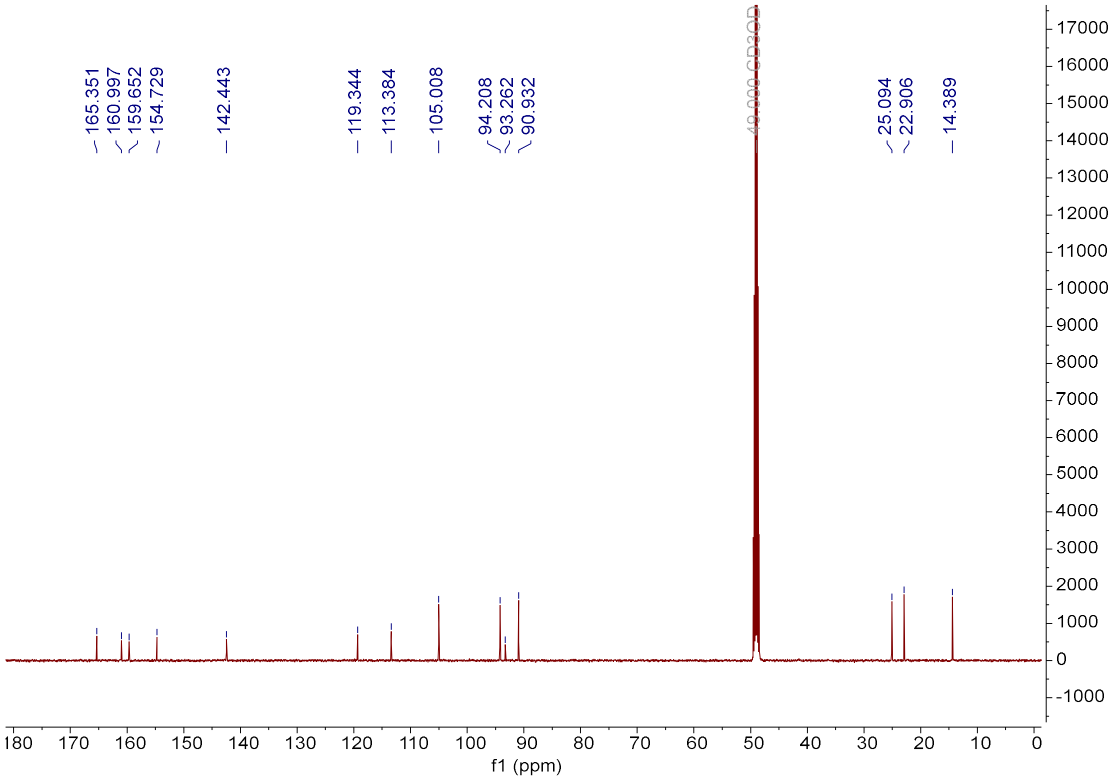

Figure S29. ^{13}C NMR (125 MHz, CD_3OD) spectrum of **4**.

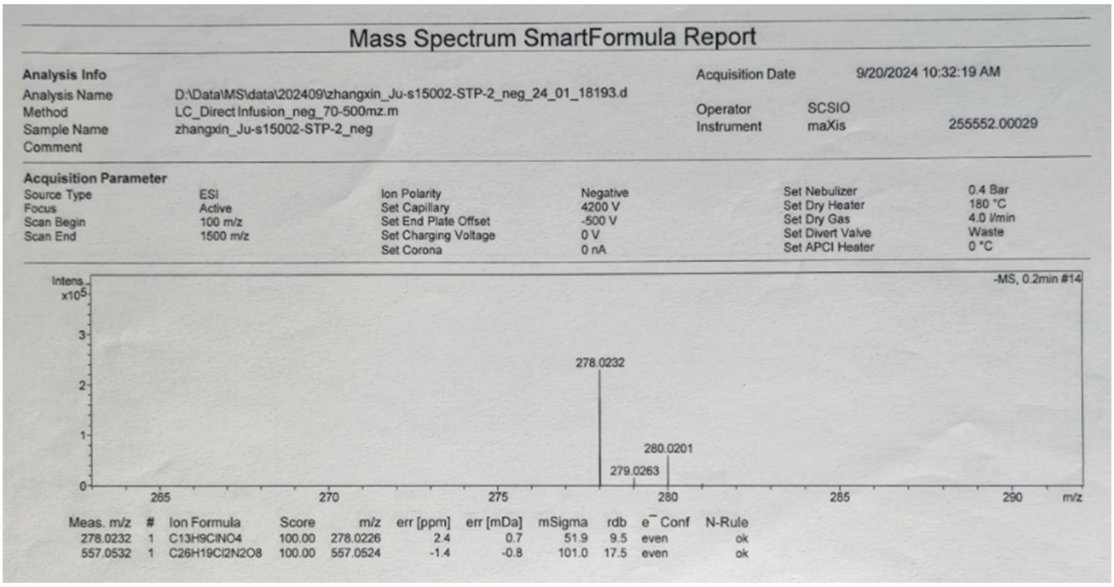

Figure S30. HRESIMS spectrum of **5**.

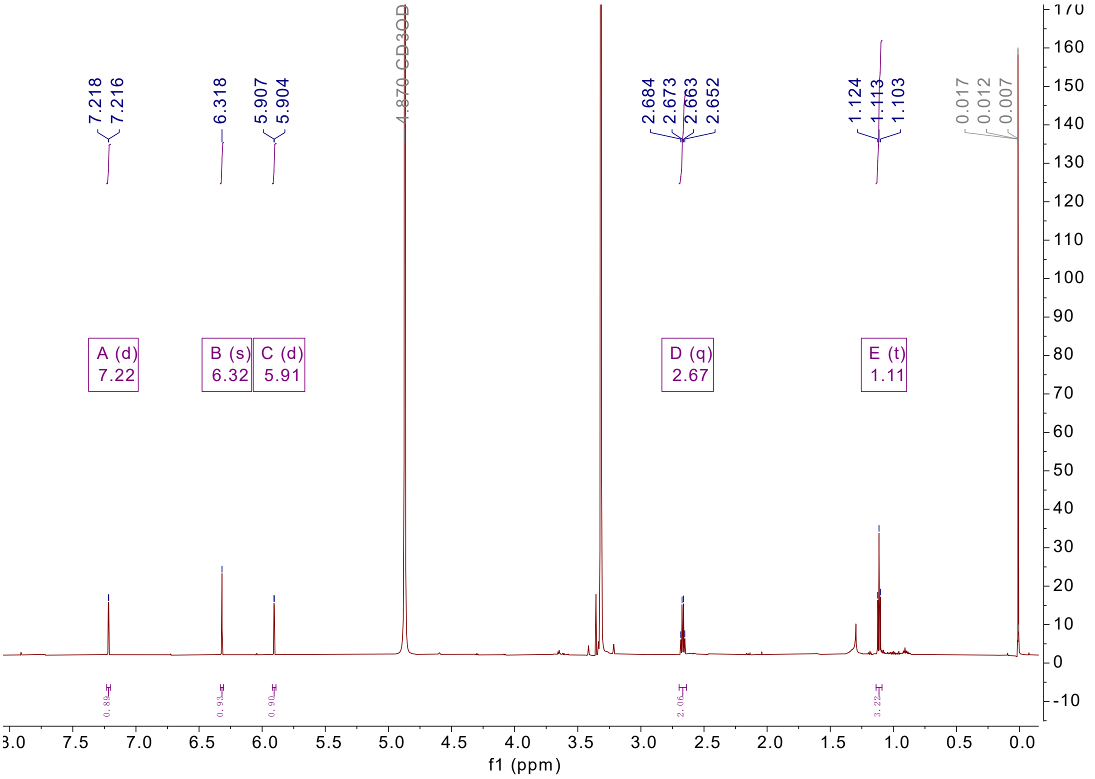

Figure S31. ^{1}H NMR (700 MHz, CD_3OD) spectrum of **5**.

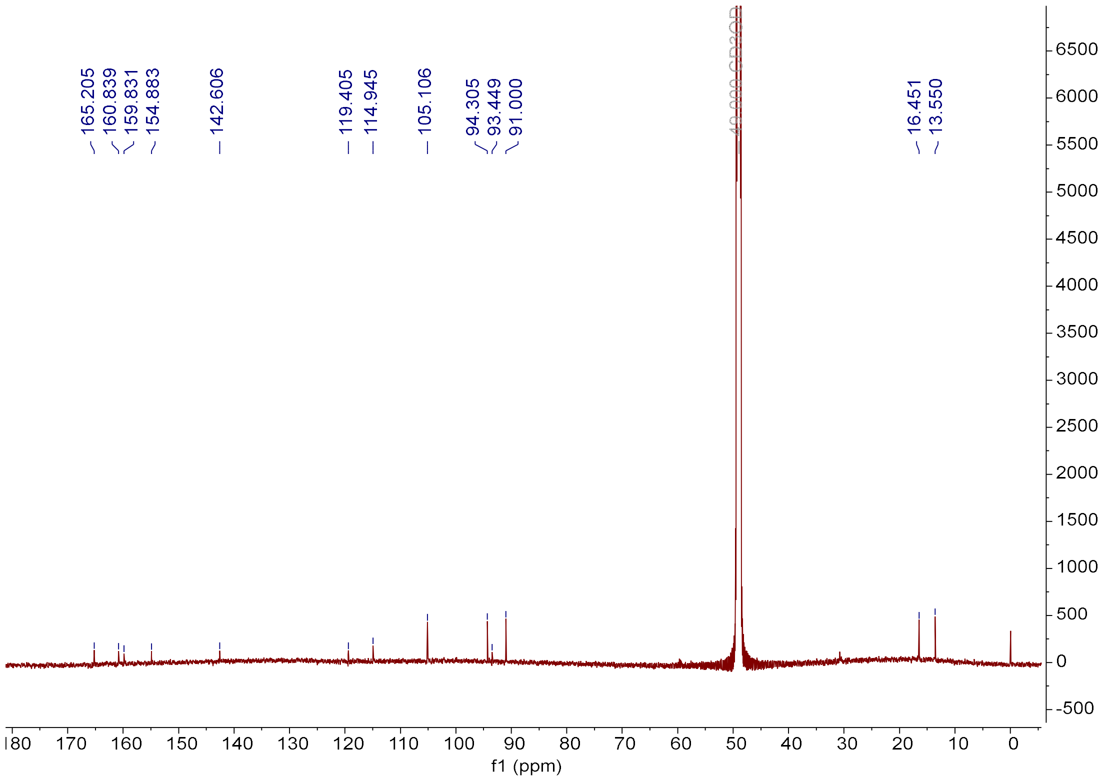

Figure S32. ^{13}C NMR (175 MHz, CD_3OD) spectrum of **5**.

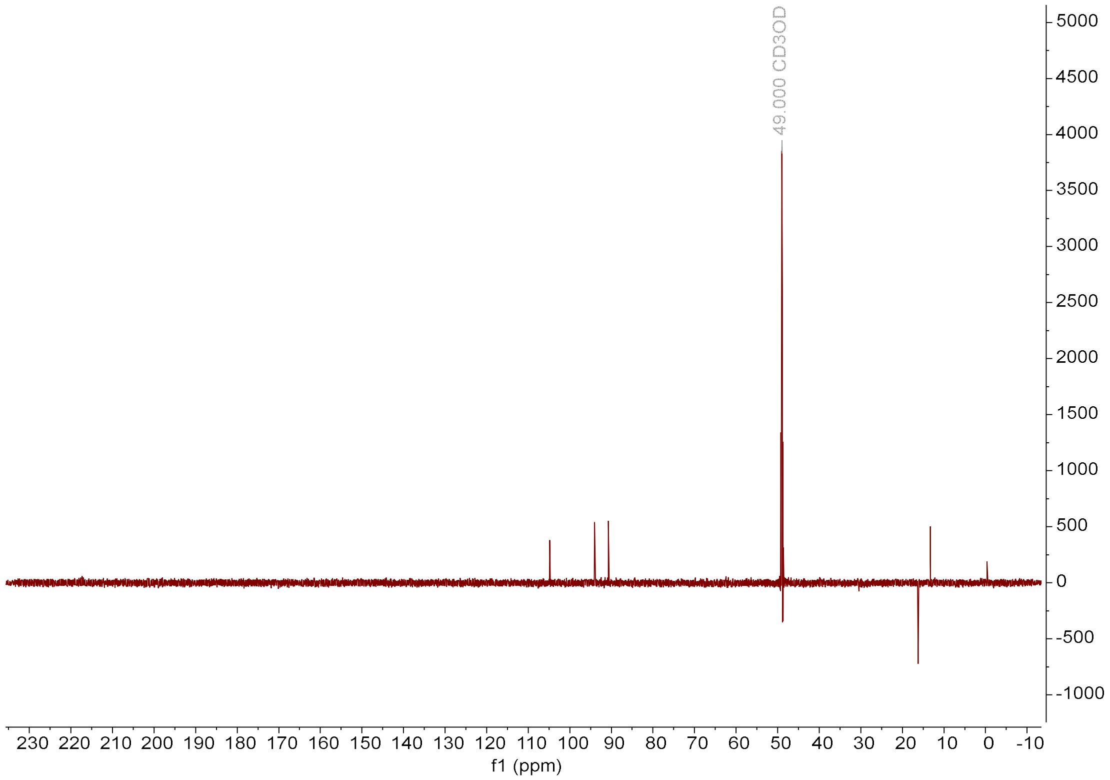

Figure S33. DEPT 135 (175 MHz, CD_3OD) spectrum of **5**.

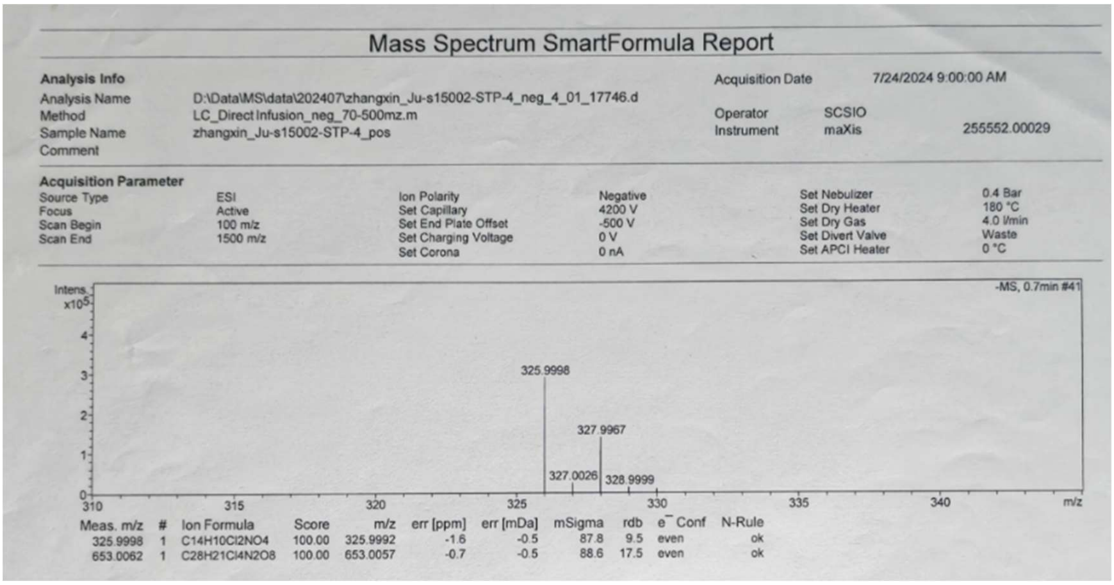

Figure S34. HRESIMS spectrum of **6**.

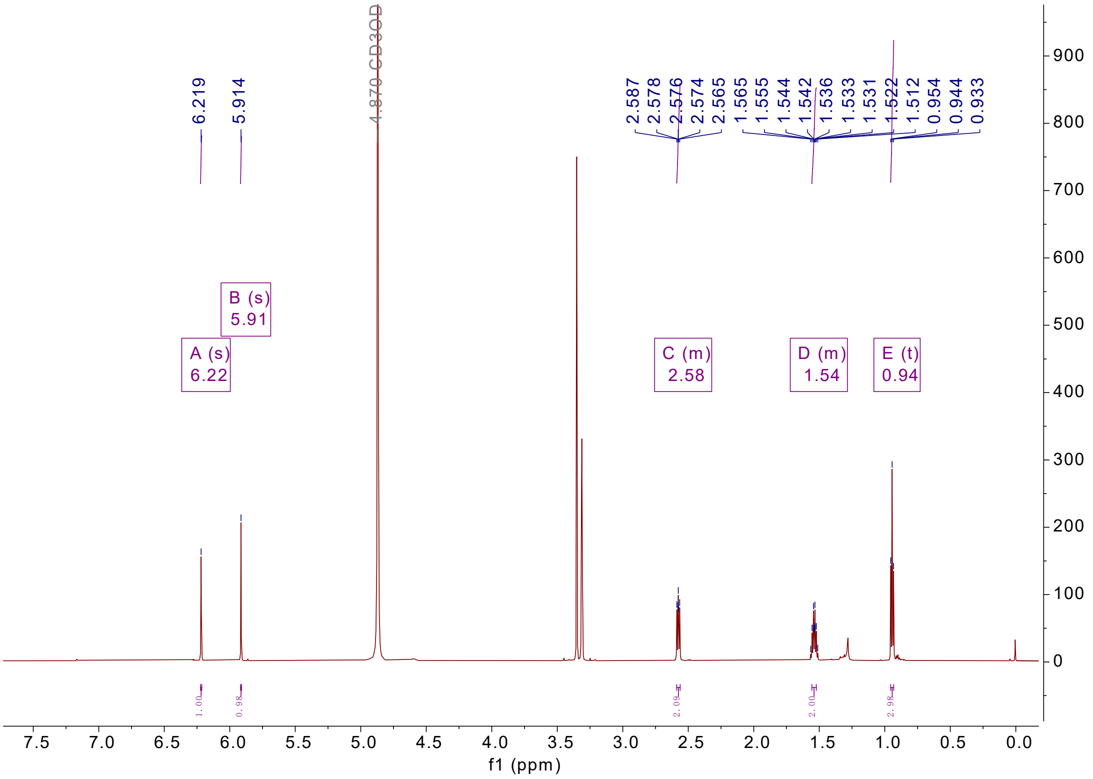

Figure S35. ^{1}H NMR (700 MHz, CD_3OD) spectrum of **6**.

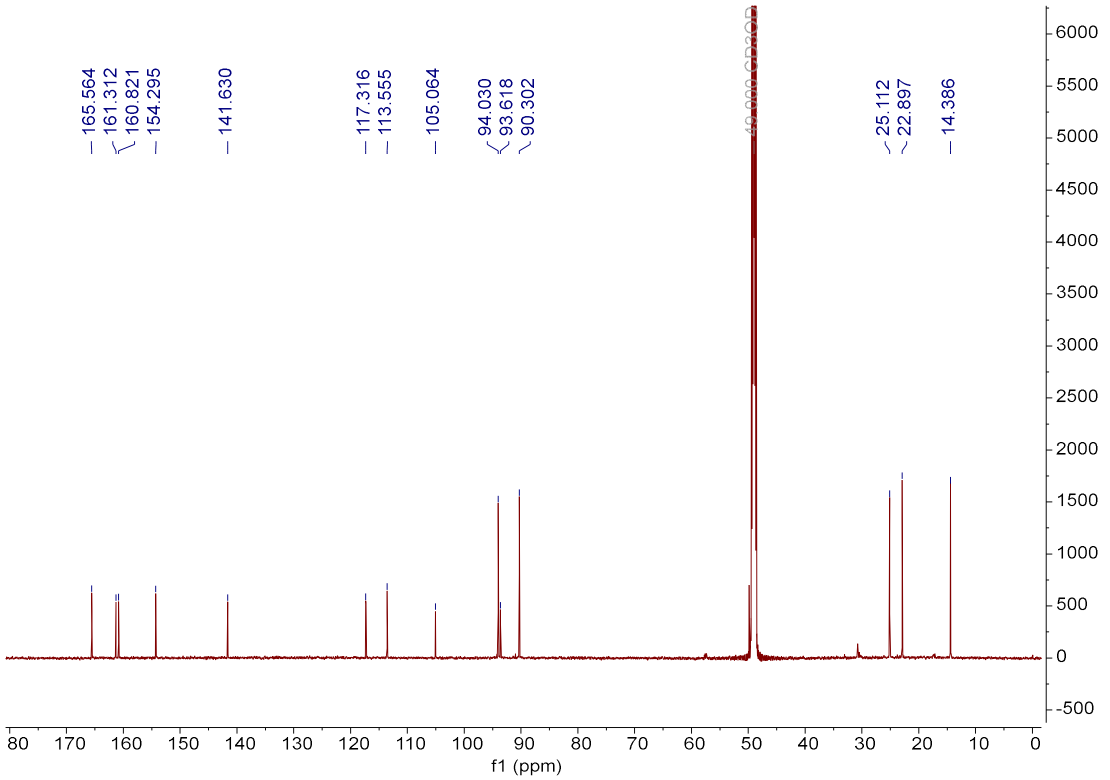

Figure S36. ^{13}C NMR (175 MHz, CD_3OD) spectrum of **6**.

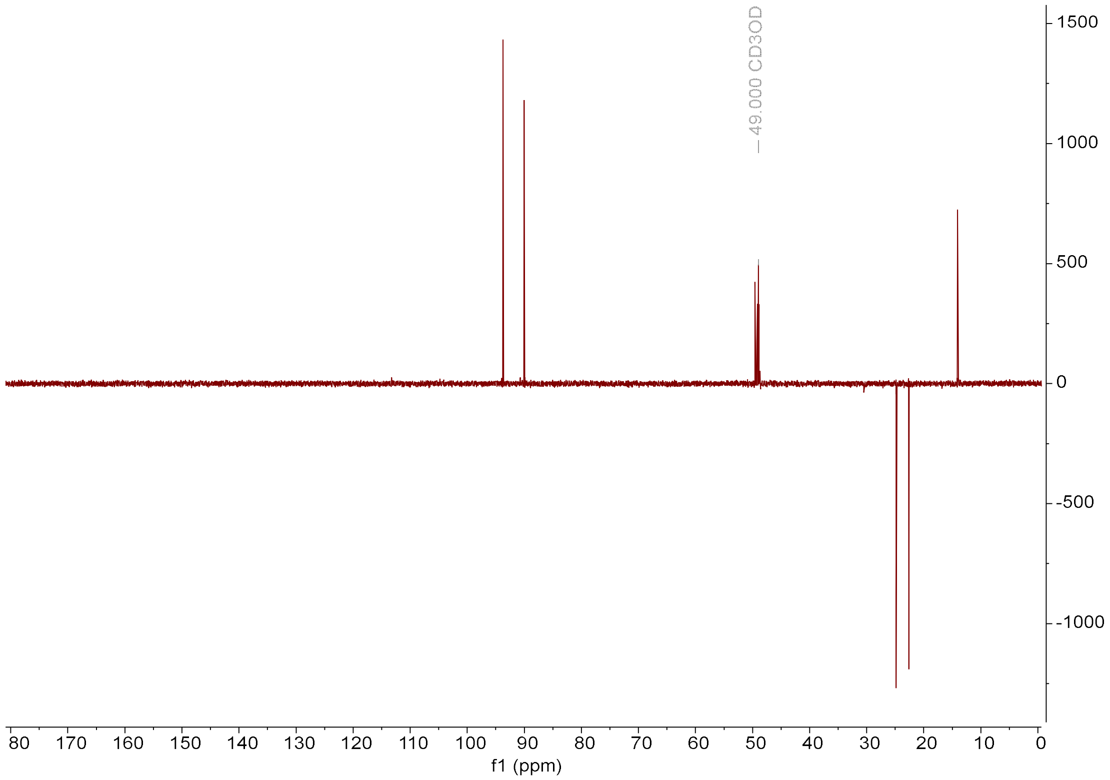

Figure S37. DEPT 135 (175 MHz, CD_3OD) spectrum of **6**.

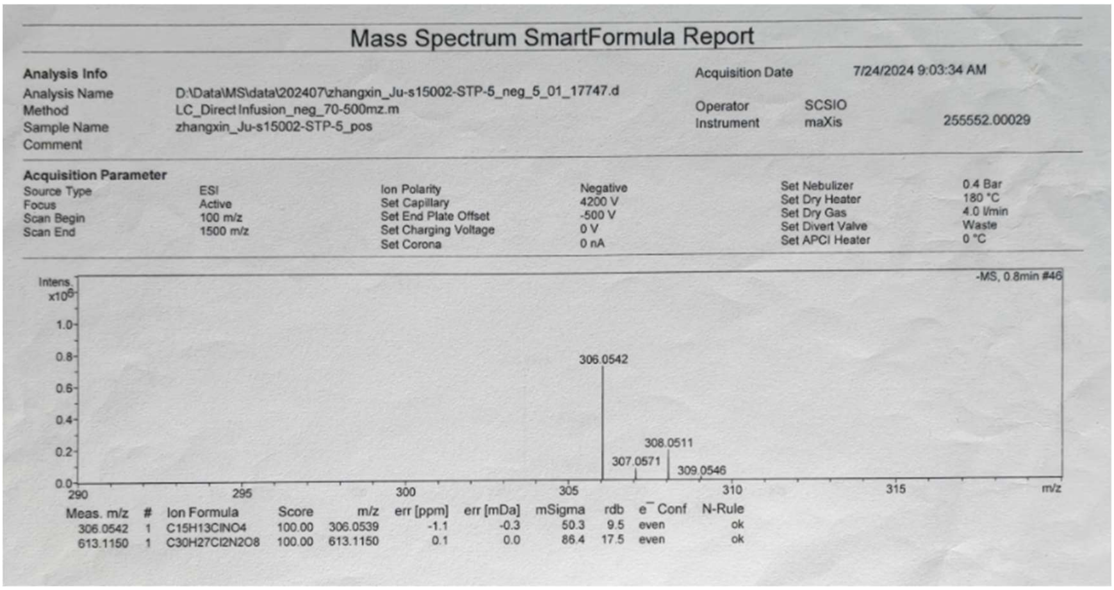

Figure S38. HRESIMS spectrum of **7**.

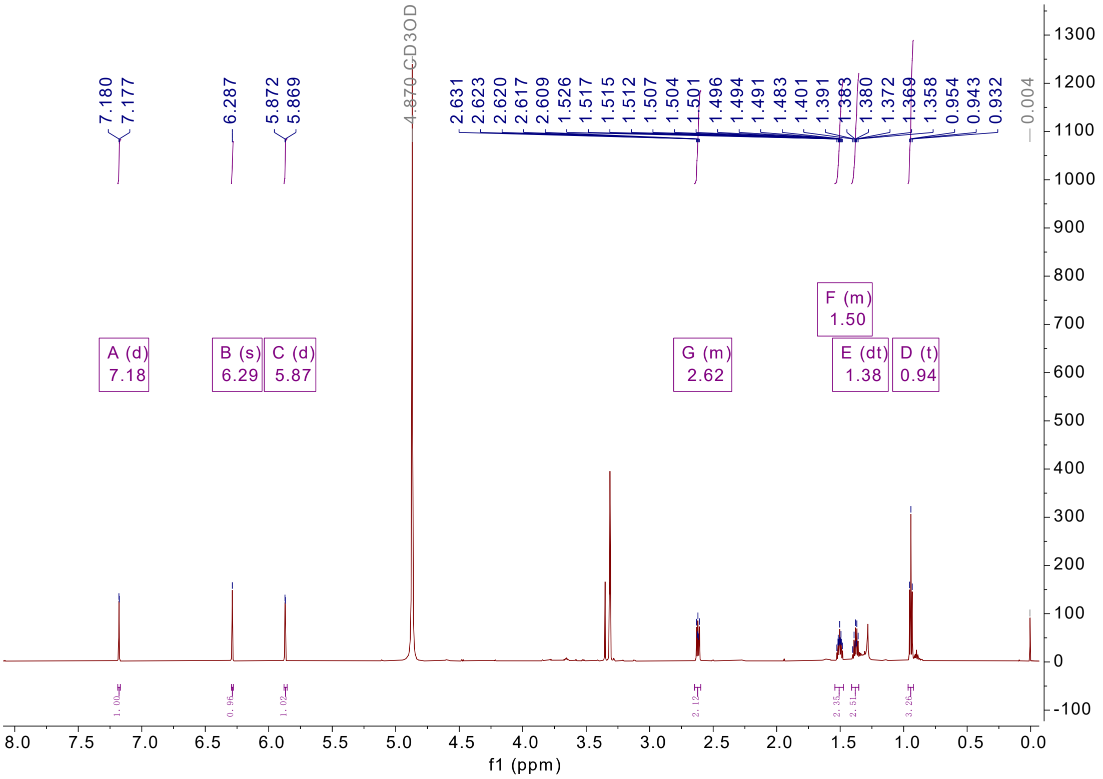

Figure S39. ^{1}H NMR (700 MHz, CD_3OD) spectrum of **7**.

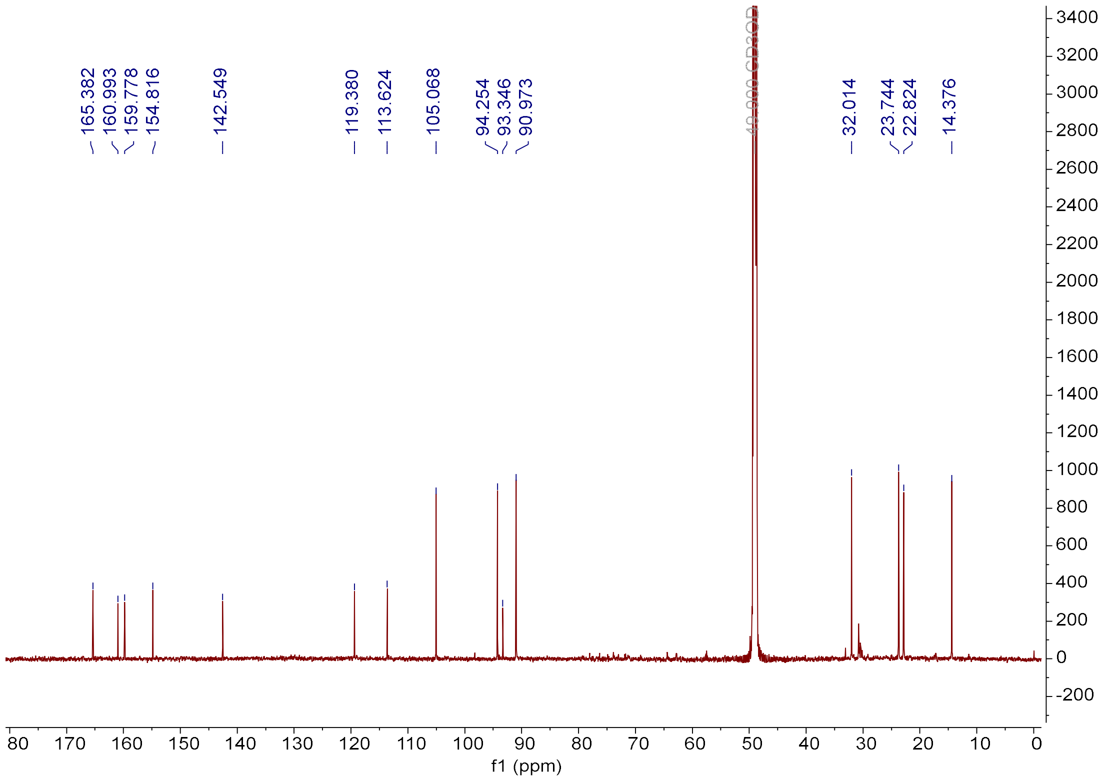

Figure S40. ^{13}C NMR (175 MHz, CD_3OD) spectrum of **7**.

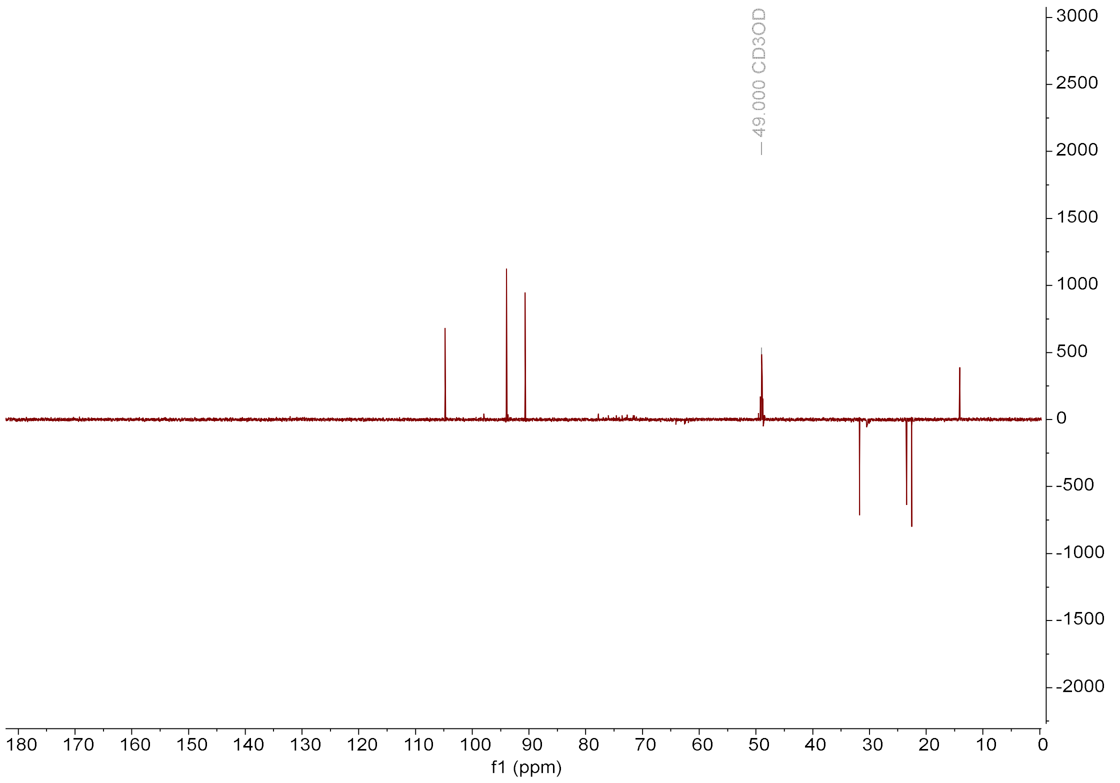

Figure S41. DEPT 135 (175 MHz, CD_3OD) spectrum of **7**.

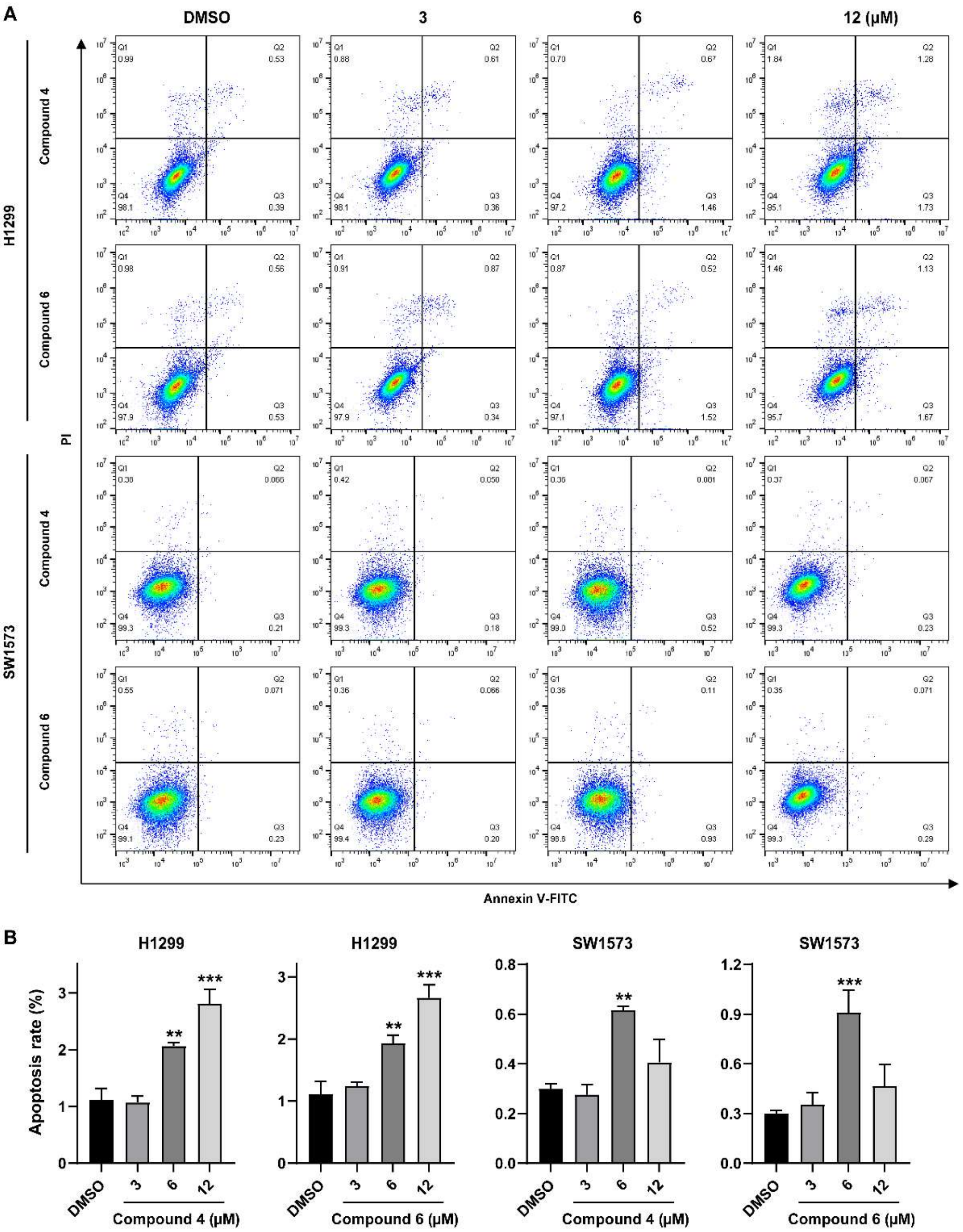

Figure S42. Compounds **4** and **6** induced a slight apoptosis in lung cancer cells. **(A)** Effects of compounds 4 and 6 on apoptosis of H1299 and SW1573 were analyzed by flow cytometry using Annexin V/PI staining. **(B)** Quantization for Fig. S43A. Data was presented as Mean ± SD, and significance was analyzed by student's *t*-test: *, $P < 0.05$; **, $P < 0.01$; ***, $P < 0.001$.
